# A Multisite Microkinetic Framework for Describing Interfacial Kinetics in Dry Methane Reforming (DRM) over Ni-CeO_2_ Catalysts

**DOI:** 10.1021/acscatal.5c07743

**Published:** 2026-01-14

**Authors:** Nirenjan Shenoy Padmanabha Naveen, Kerry M. Dooley, Michael J. Janik, Gina Noh, Konstantinos Alexopoulos

**Affiliations:** † Department of Chemical Engineering, 8082 The Pennsylvania State University, University Park, Pennsylvania 16802, United States; ‡ Department of Chemical Engineering, 5779 Louisiana State University, Baton Rouge, Louisiana 70803, United States

**Keywords:** interfacial reactions, microkinetic modeling, multisite, methane dry reforming, supported Ni catalysts, oxide-metal interface, ceria support

## Abstract

Oxide-supported Ni catalysts are widely employed for the dry reforming of methane (DRM), where the metal–support interface plays a pivotal role in mediating interfacial O-transport and H-spillover reactions. In this work, a multisite microkinetic model is developed for the Ni-CeO_2_ system to elucidate how interfacial processes govern the overall DRM activity and/or selectivity. Kinetic parameters for the model are obtained from density functional theory (DFT), and they are adjusted to ensure thermodynamic consistency, while geometric parameters are derived from an assumed catalyst model. Analyses of reaction orders reveal mixed dependencies of DRM rate on CH_4_ and CO_2_ pressures, depending on the prevailing kinetic regime. Global sensitivity analysis (Sobol) identifies the Ni nanoparticle radius (*r*_m_) as a dominant geometric parameter controlling the overall rate. Degree of rate control (DRC) analysis shows that CH_4_ activation is rate-determining for small Ni nanoparticles, while O-transport becomes limiting at a larger *r*_m_, indicating a transition to deactivation-prone regimes. The model captures this transition without explicitly incorporating coking pathways, demonstrating its robustness in representing interfacial effects. This multisite model establishes a mechanistic framework for examining transport across metal–support boundaries and serves as a predictive tool for studying interface-mediated reaction systems.

## Graphical abstract

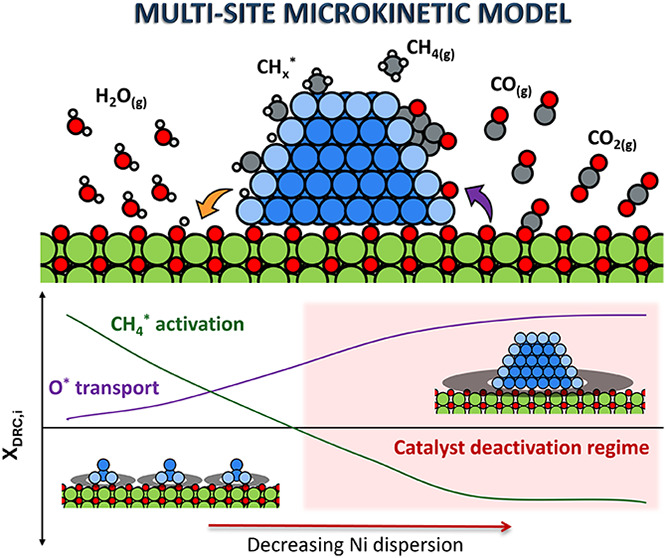

## 1 Introduction

Supported metal catalysts are prevalent in heterogeneous catalysis. Non-transition-metal oxide supports primarily disperse active metal species and seldom participate in the catalytic cycle. In contrast, for transition metal oxide supports, metal/support interfaces provide active sites and reaction paths that are unattainable on a metal or support alone. Strong metal–support interactions (SMSI)^1^ provide synergistic behavior by modulating electronic structure, stabilizing reactive intermediates, and allowing interfacial species transport to enhance catalyst activity, stability, and selectivity.^2^

Support oxygen vacancy formation energies are often lower at the metal particle interface than on pure oxide sites due to the high reactivity of interfacial O atoms.^2–5^ This effect is critical for reactions that follow the Mars-van Krevelen mechanism,^2,6–9^ as the reactive O atoms actively participate in oxidation reactions. Interfaces perturb the electronic environment of the neighboring metal atoms and can enable facile activation of stable bonds in gases like CH_4_, with studies showing that C–H bond activation barriers are lower at the interface^10–14^ than on noninterfacial metal sites. Depending on the support oxide reducibility, interfacial O species can also be transported to the metal particle, thereby driving O-dependent reaction paths.^15–17^ Ni–CeO_2_ catalysts exhibit such interfacial effects, conferring high reactivity and stability for DRM, unlike Ni–Al_2_O_3_ catalysts that deactivate via carbon deposition due to an inactive interface.^18–21^

The Ni metal, interfacial Ni–O–Ce, and CeO_2_ surface sites can all play a mechanistic role in the DRM on Ni–CeO_2_ catalysts. Ni and Ni–O–Ce sites activate CH_4_, causing CH_x_* dehydrogenation followed by H_2_ formation. Being a reducible support, CeO_2_ exhibits exceptional oxygen storage capacity,^22^ and promotes O-transport to the Ni metal to facilitate oxidation of surface C* or CH_x_* species to CO. This self-cleaning behavior of the oxide-metal interface is well recognized in DRM, as Ni catalysts are often prone to carbon deposition.^2^ The resulting oxygen vacancies on the support or interface readily activate CO_2_, with a low energy barrier,^23^ regenerating the active O species, and producing CO. Despite offering such advantages in DRM, the interface can also allow H-spillover from Ni to CeO_2_, leading to consumption of H* species to form H_2_O (leaving an O vacancy), effectively facilitating the reverse water–gas shift (rWGS) reaction and reducing overall product selectivity (H_2_:CO ratio).^24–27^ Modulating the metal/metal oxide interface to suppress H-spillover while promoting O-transport is essential for maximizing DRM performance.

Multiple experimental studies have designed Ni-based multicomponent catalysts by modulating the composition of reducible oxide (CeO_2_, La_2_O_3_, ZrO_2_)^28–31^ and irreducible oxide (Al_2_O_3_, SiO_2_),^28,32–34^ resulting in semireducible catalysts that are active and highly selective. However, developing a comprehensive understanding of the mechanistic role of interfacial events is challenging. The structural complexity and heterogeneity of interfaces, along with their dynamic nature, make them difficult to characterize using conventional experimental techniques.^35,36^ Traditional spectroscopic techniques lack the spatial and temporal resolution to isolate interfacial contributions.^35,36^ Furthermore, the metal–support contact geometry, particle size, and structure critically influence the interfacial activity, making mechanistic studies highly system-dependent.

Computational models can be useful to investigate these effects. Density Functional Theory (DFT) has proven to be valuable in exploring interfacial phenomena. Several studies have compared the mechanism and reaction energetics of CH_4_/CO_2_ activation on metal, support, and interfacial sites.^11–14,26^ Yet, a limited number of studies probe the interfacial species transport or couple DFT energetics with elementary kinetic models that consider interfacial structure. Foppa et al. developed a comprehensive DFT-based microkinetic model (MKM) for DRM and WGS, showing that the Ni–Al_2_O_3_ interface is ineffective in altering DRM reactivity and selectivity.^37^ Jiao, and Wang also proposed a multisite MKM for Ni–ZrO_2_ catalysts, demonstrating how interfacial effects influence carbon deposition under applied electric fields.^38^ We seek to develop a multisite interfacial MKM for DRM that can include interfacial species transport and explore the impact of particle size/interfacial structure on reaction kinetics.

In this study, we advance beyond prior efforts using DRM and rWGS to examine the role of interfacial reactions. A multisite MKM is developed using Ni–CeO_2_ as the representative catalyst. Kinetic parameters are obtained from density functional theory (DFT) literature and refined using Sequential Least Squares Programming (SLSQP) optimization, enabling minimal adjustments to kinetic parameters while strictly satisfying thermodynamic consistency. Catalyst geometry effects, specifically the influence of metal particle size and interfacial radius on reaction pathways and rate-determining steps, are considered. In addition to predicting rate and selectivity behavior, the model captures reaction dynamics under deactivation-prone regimes, such as coking at high CH_4_/CO_2_ molar ratios and large Ni nanoparticle sizes. The model also reproduces operating-condition-dependent reaction orders in CH_4_ and CO_2_, consistent with the experimental observations. Altogether, this work introduces a versatile and thermodynamically grounded modeling framework that bridges interfacial chemistry, geometric control, and operating condition sensitivity, offering predictive insights and design principles for interface-engineered, deactivation-resistant catalysts.

## 2 Computational Details

This section comprehensively details model development for the multisite MKM framework. As the name suggests, a multisite MKM requires definition of each type of site, the areal density of each type of site, and the set of reactions that can occur on combination of these sites. The “multisite” aspect of this study is based on a Ni-CeO_2_ catalyst model, with two broad classifications of sites: (1) metal and (2) support. The interfacial region is embedded within these sites. Section 2.1 provides information about the nomenclature of these sites, along with their respective site quantification. Section 2.2 contains details on the elementary reactions included in the MKM, along with unique reaction paths for DRM/rWGS. Kinetic parameters for many of these reactions are obtained from different literature sources and adjusted to achieve thermodynamic consistency, as described in Section 2.3. Rate expressions for elementary reactions are derived using the adjusted kinetic parameters. The mean-field approach is utilized, with unique definitions for interfacial reaction rates. Section 2.4 provides details on the formulation of rate expressions along with the model solution framework. A list of abbreviations and notations used in this work is included in Table 1.

**Table 1:** Abbreviations and Notation

shortform	definition
DRM	dry reforming of methane
rWGS	reverse water-gas shift
MKM	microkinetic model
DFT	density functional theory
R*_i_*	reaction *i*
DRC	degree of rate control (analysis)
X_DRC,*i*_	degree of rate control of reaction *i* (value)
*r*_m_	radius of Ni nanoparticle (nm)
*r*_int_	interfacial radius (nm)
p_j_	partial pressure of species *j* (bar)
θ_k_	steady-state coverage of adsorbate species *k* (ML)

### 2.1 Catalyst Model: Site and Geometric Parameters

The catalyst model can be visualized as uniform hemispherical Ni nanoparticles deposited on a CeO_2_ surface. The hemispherical shape is a reasonable idealization, as it closely matches the equilibrium spherical-cap geometry for typical metal oxide adhesion strengths.^39^ Ni atoms on the nanoparticle are approximated to replicate the fcc-Ni(111) type arrangement. A schematic representation of the cross-sectional and top view of a Ni particle with a surrounding support is presented in Figure 1. Surface Ni sites (also the active metal sites), represented as “*”, are shaded in light blue, with the subsurface and bulk atoms displayed in dark blue circles. The total number of Ni sites (N_Ni,tot_) is computed using experimental Ni loading per gram of total catalyst (site calculations are shown in Section S1). The fraction of surface sites (NS_Ni,tot_) is calculated from the curved surface area of the nanoparticle. Ni sites located on the periphery of the nanoparticle are deemed interfacial metal sites and are quantified from the circumferential length. All surface Ni sites participate in non-interfacial reactions, while only interfacial Ni sites catalyze H-spillover reactions.

**Figure 1:**
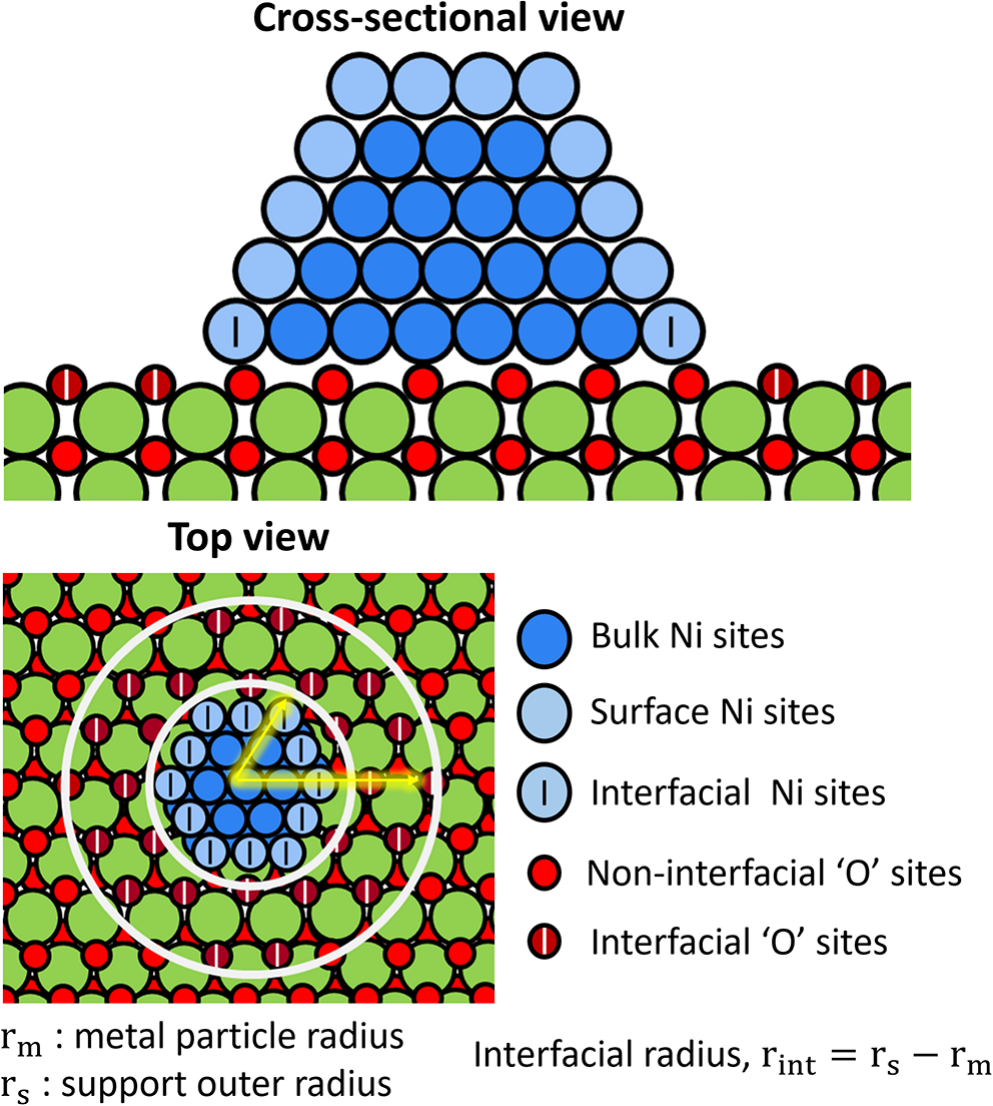
Schematic representation of the catalyst model along with definitions of the metal and support region.

CeO_2_(111) was selected as the support model because it is the most stable low-index facet and is known to be most abundant under high-temperature conditions relevant to DRM.^28,40,41^ The support has two distinct active sites on its surface: (1) lattice O represented as “O^v^”, and (2) vacancy sites represented as “^v^”; these sites together constitute the total support active sites (NS_s, tot_; described in detail in the SI, Section S1). They are quantified from experimental nitrogen physisorption isotherms, interpreted using BET analysis.^28^ The interfacial radius, *r*_int_ (“doughnut” region around a metal particle, Figure 1), defines the interfacial support region. All support sites can participate in any support-relevant surface reactions, with the O^v^ sites in the interfacial (“doughnut”) region exclusively responsible for the interfacial O-transport.

Although interfacial sites in both the metal and support are quantified separately from the respective total sites, they are not treated as energetically distinct sites in the elementary reactions. Thus, the unique contribution of the interfacial reaction rates arises solely from the quantified interfacial site densities. The total number of interfacial metal sites is fixed for a given nanoparticle size as it is derived from the nanoparticle circumference. On the other hand, the total interfacial support sites depend on the interfacial radius, *r*_int_, which is treated independently of *r*_m_. Unless otherwise noted, *r*_int_ is set to 0.38 nm, corresponding to the distance between neighboring oxygens on the geometry optimized CeO_2_(111) surface. This distance represents the first support O-coordination shell surrounding the Ni nanoparticle.

### 2.2 Elementary Reactions

A total of 17 elementary reactions are considered, with some of them occurring exclusively on metal or support sites and interfacial reactions involving both types of sites. Table 2 contains the reactive species nomenclature. Reactions pertaining to both DRM and rWGS are included in the model (Table 3); the complete reaction scheme is presented in Figure 2.

**Table 2:** Nomenclature for Empty Active Sites/Adsorbed Species on Active Sites

*	bare Ni (metal) active site
X*	species X adsorbed on a bare Ni (metal) site
^v^	support site
X^v^	species X adsorbed on a support site

**Table 3:** Elementary Reactions Considered in the MKM, Noting Sets of Elementary Steps, and Their Respective Stoichiometric Numbers That Would Constitute a Complete DRM (D1 or D2) or rWGS (W1 or W2) Path

reaction #, *R_i_*	reaction	D1	D2	W1	W2
R1	R1 $C{H_{4}}_{\left(g\right)}+*↔CH_{4}^{*}$	1	1	0	0
R2	R2 $CH_{4}^{*}+*↔CH_{3}^{*}+H^{*}$	1	1	0	0
R3	R3 $CH_{3}^{*}+*↔CH_{2}^{*}+H^{*}$	1	1	0	0
R4	R4 $CH_{2}^{*}+*↔{\mathrm{CH}}^{*}+H^{*}$	1	1	0	0
R5	R5 $CH^{*}+*↔C^{*}+H^{*}$	1	0	0	0
R6	R6 $2H^{*}↔{H_{2}}_{\left(g\right)}+2^{*}$	2	2	–1	–1
R7	R7 $CH^{*}+O^{*}↔\mathrm{CH}O^{*}+*$	0	1	0	0
R8	R8 $\mathrm{CH}O^{*}+*↔CO^{*}+H^{*}$	0	1	0	0
R9	R9 $C^{*}+O^{*}↔CO^{*}+*$	1	0	0	0
R10	R10 $CO^{*}↔CO_{\left(g\right)}+*$	1	1	0	0
R11^a^	R11 $O^{v}+*↔O^{*}+v$	1	1	0	–1
R12	R12 $C{O_{2}}_{\left(g\right)}+v↔CO_{2}^{v}$	1	1	1	0
R13	R13 $CO_{2}^{v}↔{\mathrm{CO}}_{\left(g\right)}+O^{v}$	1	1	1	1
R14	R14 ${{\mathrm{CO}}_{2}}_{\left(g\right)}+O^{v}↔CO_{3}^{v}$	0	0	0	1
R15^a^	R15 $CO_{3}^{v}+*↔CO_{2}^{v}+O^{*}$	0	0	0	1
R16^a^	R16 $H^{*}+O^{v}↔{\mathrm{OH}}^{v}+*$	0	0	1	1
R17^a^	R17 ${\mathrm{OH}}^{v}+H^{*}↔H_{2}O_{\left(g\right)}+v+*$	0	0	1	1

a Denotes interfacial reactions.

**Figure 2:**
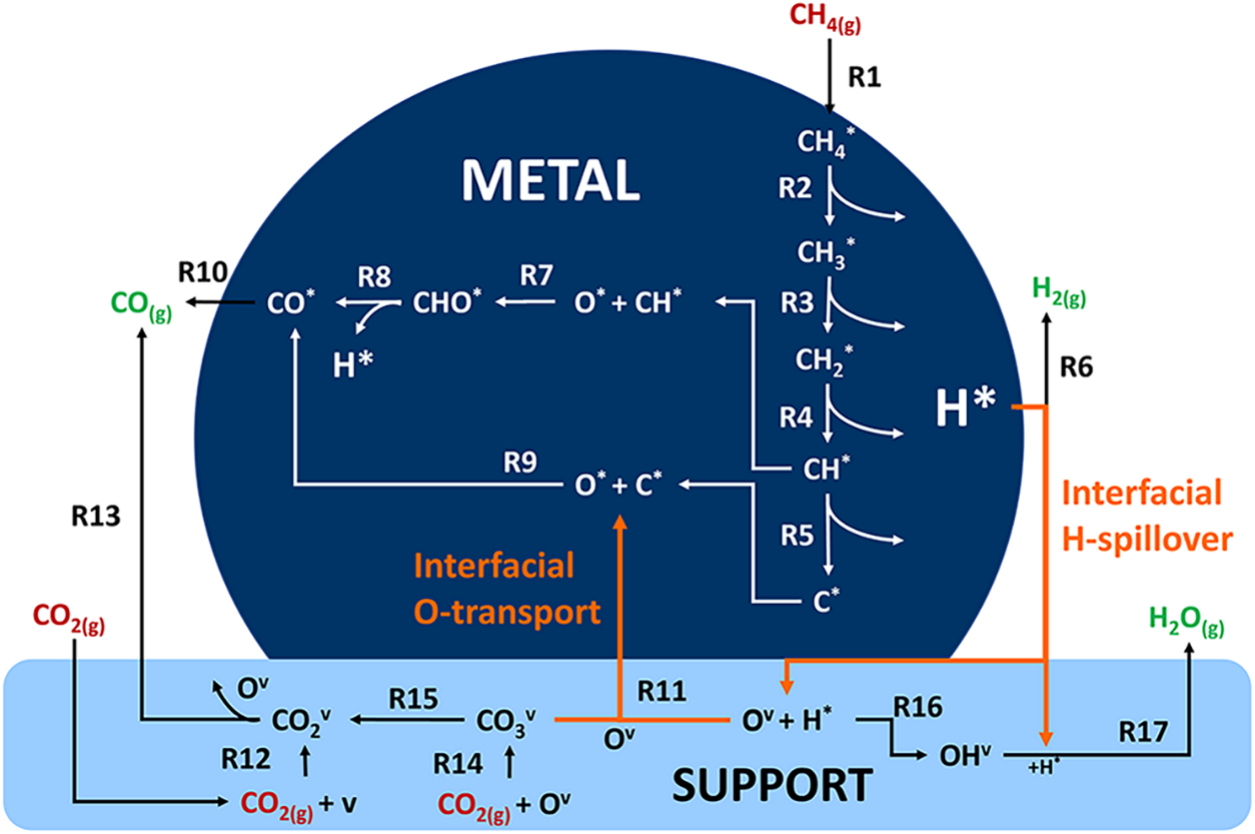
Schematic representation of elementary reactions included in the MKM, species in red and green fonts represent reactants and products, respectively.

Activation of the C–H bond in CH_4(g)_ on Ni has lower transition state energies than on CeO_2_;^11^ thus, all elementary steps for CH_x_* dehydrogenation ([Disp-formula eq2]–[Disp-formula eq5]) are assumed to occur on Ni sites. Prior mechanistic studies report that interfacial sites can further lower the C–H bond activation barriers; however, the activation still proceeds through the conventional CH_3_*-forming pathway.^10–14^ Pathways involving oxygenated species remain energetically less favorable.^42^ Thus, the direct participation of the O*/OH* species in CH_4_ activation is excluded from the reaction set.

Two metal-based CO_(g)_ formation mechanisms are considered: (1) direct “C*” oxidation to “CO*” via [Disp-formula eq9], or (2) sequential “CH*” oxidation to “CHO*” ([Disp-formula eq7]), followed by “CO*” formation ([Disp-formula eq8]), leading to two distinct DRM pathways, D1 and D2, respectively (Table 3). “CH*” and “C*” oxidation reactions via “OH*” species were considered initially but removed for model simplicity because reaction path analysis indicated their role in CO_(g)_ formation was negligible. Elementary steps forming C–C bonds were not included, although high C* coverages that may lead to the formation of deactivating carbonaceous deposits are discussed (vide infra, Section 3.2).

CeO_2_ has excellent oxygen storage capacity, which enables facile activation of CO_2(g)_ compared to pure metals;^23,43,44^ thus, it is assumed that CO_2_ activation occurs only on the support. CO_2(g)_ is converted to CO_(g)_ via two mechanisms: (1) adsorption on “^v^” sites followed by direct CO_(g)_ formation ([Disp-formula eq12], 13), or (2) adsorption on “O^v^” sites to form “CO_3_^v^” species ([Disp-formula eq14]), which react at the interface to form “O*” and “CO_2_^v^” ([Disp-formula eq15]) followed by CO_(g)_ formation ([Disp-formula eq13]). These two mechanisms lead to unique rWGS paths W1 and W2. In agreement with the literature,^45^ our DFT calculations show that CO binds only weakly on stoichiometric CeO_2_(111) (Δ*E*_ads_ = −0.06 eV), forming a physisorbed species that remains kinetically irrelevant under low-conversion DRM conditions; this intermediate is thereby excluded for model compactness. H-assisted CO_2(g)_ activation paths on the metal nanoparticle are not included as they have huge activation barriers and play a negligible role in CO_(g)_ formation.^46,47^

Two interfacial processes are explicitly included: (1) O-transport from support to the metal particle (via “O^v^” ([Disp-formula eq11]) or “CO_3_^v^” ([Disp-formula eq15]) species), forming O* that is then responsible for “C*” oxidation and CO_(g)_ formation on the metal, (2) H-spillover from metal to support (via “O^v^” ([Disp-formula eq16]) and “OH^v^” ([Disp-formula eq17]) species), responsible for H_2_O_(g)_ formation on the support. The reaction 2OH^v^ ↔ H_2_O_(g)_ + O^v^ + v was not included as an alternative pathway for H_2_O formation, as it is a linear combination of the reactions 16 and 17.

### 2.3 Kinetic Parameter Estimation and Validation

All the elementary reactions (*i*) are considered reversible, with forward or reverse (denoted as *j* = *f* and *r*, respectively) rate constants (*k*_*i*,*j*_) determined using the Eyring-Polanyi Equation:^48^

1 $$k_{i,j}=A_{i,j}\cdot \exp\left(-\frac{\Delta H_{j}^{‡}}{\mathrm{RT}}\right)$$

where *A*_*i*,*j*_, the preexponential factor for reaction *i*, is given by

2 $$A_{i,j}=\left(\frac{k_{B}\cdot T}{h}\right)\cdot \exp\left(\frac{\Delta S_{j}^{‡}}{R}\right)$$

and *k*_B_ is the Boltzmann constant, *T* is the absolute temperature, *h* is Planck’s constant, and *R* is the gas constant. Activation enthalpies (Δ*H*_j_^‡^) and preexponential factors (*A_i_*_,_ *_j_*) for [Disp-formula eq10], 13, 14, 16, and 17 were determined using DFT calculations for this work (Section 2.3.1), and the remainder were obtained from DFT literature references (reported in the SI, Section S2).

Catalyst models used for DFT calculations in the literature references include single Ni atoms or small Ni clusters supported on CeO_2_, an extended Ni(111) surface, and an extended CeO_2_(111) surface. As a result, the values for even a single parameter can vary by orders of magnitude. For example, values of Δ*H*_f_^‡^ for the first C–H bond activation ([Disp-formula eq2], Table 3), which is generally considered to be the rate-determining step (RDS) in DRM,^26,38,49–51^ range from 9 to 114 kJ mol^–1^ (tabulated in Table S1). Similar differences were also observed for Δ*H*_f_^‡^ values for other example reactions such as [Disp-formula eq6] (74–195 kJ mol^–1^) and 13 (126–260 kJ mol^–1^) (Tables S2 and S3, respectively). The choice of a representative value for each reaction is extremely challenging, and this is exacerbated by the heterogeneity of the settings used in the DFT calculations. Thus, we strive to select parameters guided by physical constraints and test the sensitivity of the observed kinetics to the choice of parameters.

Thermodynamic consistency was utilized as a guiding criterion for the kinetic parameters. First, NIST-reported^52^ values of gas-phase Δ*G*_DRM_, Δ*G*_rWGS_ were evaluated. From the broad range of kinetic parameters for each elementary reaction, the values that yielded the lowest deviations from the NIST data were selected for every distinct DRM and rWGS pathway. Deviations were taken to be the difference between DFT-calculated and NIST-reported Δ*G*_DRM_ or Δ*G*_rWGS_ values (magnitudes of error compared to gas-phase thermochemical values presented in the SI, Section S2B). Next, we made modest adjustments to the DFT-calculated Δ*H*_rxn_ and Δ*S*_rxn_, according to their magnitude of error contribution.

The concept of incorporating parameter adjustments to attain thermodynamic consistency has previously been implemented in existing microkinetic modeling studies for a variety of reaction systems.^53–55^ There are many ways to implement the corrections given the total number of kinetic parameters in the current model. In this work, a Sequential Least Squares Programming (SLSQP) method was chosen because it provides minimal near-global corrections, given a set of physical constraints (additional details in the SI, Section S2B). Through this, we demonstrate that it is not always necessary to recompute an entire kinetic data set when dealing with well-established reaction systems. Instead, gas-phase thermodynamic consistency can still be rigorously achieved through global optimization, even when values from multiple literature sources. This highlights the broader utility of the approach for constructing internally consistent microkinetic models without requiring a complete in-house DFT data set.

We note that despite our implementation of a systematic methodology to adjust parameters consistently, the resulting observations are sensitive to these kinetic parameters. Thus, we substantiate the model-predicted results against published experimental studies wherever possible and evaluate the sensitivity of the results to selected parameters.

#### 2.3.1 Electronic Structure Methods

Reaction energies and activation barriers for [Disp-formula eq10], 13, 14, 16, and 17 were computed using DFT calculations, using the Vienna *ab initio* Simulation Package (VASP).^56^ For [Disp-formula eq10], 16, and 17, a Ni_4_–CeO_2_(111) catalyst model was used, where a 4 Ni atom cluster was deposited on a 2 × 2 CeO_2_(111) slab model. For [Disp-formula eq13], a 2 × 2 CeO_2_(111) slab with one surface oxygen vacancy was used. For [Disp-formula eq14], a pure 2 × 2 CeO_2_(111) slab was used. The electronic exchange and correlation interactions were described by the generalized gradient approximation (GGA) method with the Perdew–Burke–Ernzerhof (PBE) functional.^57^ The projector augmented wave method was used to represent the core electrons,^58^ and a plane wave basis set was used to represent the valence electrons with an energy cutoff of 500 eV. The bulk CeO_2_ structure used to create the CeO_2_(111) slab is the commonly used cubic fluorite type structure, with a DFT-optimized lattice constant of 5.2 Å. A 2 × 2 × 1 Monkhorst pack grid^59^ was used to sample the Brillouin zone of the ceria slab. Electronic cycles converged with an energy difference of less than 10^–5^ eV. Structural optimizations minimized forces on all atoms below 0.05 eV Å^–1^. Spin-polarized calculations were used for systems with unpaired electrons. Slab-to-slab dipole interactions were corrected while simulating the surfaces. All isolated gas molecules were optimized with 1 × 1 × 1 *k*-point grid, in a 10 × 10 × 10 Å unit cell. DFT+U corrections were used for Ce, due to the well-established difficulties that DFT faces while representing the 4f orbitals of Ce. A *U*_eff_ value of 5 eV was used on the f orbitals of Ce, based on our previous work with ceria-based systems.^60^ DFT is also known to have difficulties representing the localized d-states of transition metals; thus, U corrections were also applied for systems containing Ni. A *U*-value of 6.4 eV^61^ was used on the d orbitals of Ni because of closer agreement between the experimental^62^ and simulated band gap (4.0 vs 3.89 eV).

Henkelman’s climbing image nudged elastic band (CI-NEB) was used to locate the transition states along the minimum energy path. Convergence was reached when the tangent force on the highest energy image, indicating the transition state, was reduced to less than 0.05 eV Å^–1^. Harmonic vibrational frequency analysis with a convergence of 1 × 10^–6^ eV for the energy was performed on this image to confirm the existence of the transition state. Initial and final states were subjected to vibrational frequency analysis for obtaining Δ*S*_rxn,Ri_, Δ*S*_f_^‡^, and Δ*S*_r_^‡^. Any low-lying vibrational modes (<50 cm^–1^) were set to 100 cm^–1^ to avoid erroneous entropic estimation.

### 2.4 Formulation of Rate Expressions and Model Solution Framework

The model is composed of 34 rate constants with corresponding preexponential factors, 10 geometric parameters (parameters in Table 4 along with Ni loading and specific surface area of the catalyst), and 6 operational parameters (p_CH_4__, p_CO_2__, p_H_2__, p_CO_, p_H_2_O_, *T*), and is employed to derive rate expressions for the elementary reactions. Site-relevant parameters are defined in Table 4. Ni content is set to 4 wt %, and the specific surface area of the support is set to 70 m^2^ g_cat_^–1^.

**Table 4:** Catalyst Geometry-Derived Site Parameters along with Their Definitions

NS_Ni,tot_	total number of surface Ni sites
NI_Ni,tot_	total number of interfacial (surface) Ni sites
NS_s,tot_	total number of support sites
NI_s,tot_	total number of interfacial support sites
Z^m,m^	number of nearest metal site neighbors to a metal site
Z^m,s^	number of nearest metal site neighbors to a support site
Z^s,s^	number of nearest support site neighbors to a support site
Z^s,m^	number of nearest support site neighbors to a metal site

A mean-field kinetics approach (MFA) is used, with rates on the metal and support defined as a function of the respective site number and a coordination number (CN), Z^i,j^.^63^ This can be further explained using an example reaction, A^a^ + B^a^ ↔ AB^a^ + ^a^. Depending on where the reaction occurs (a = metal or support), the forward rate expression is defined as below.

On the metal, the following reaction

$$A^{*}+B^{*}↔{\mathrm{AB}}^{*}+*$$

has a forward rate expression, r_f_, given by:

$$r_{f}=k_{f}\cdot {\mathrm{NS}}_{\mathrm{Ni},\mathrm{tot}}\cdot \theta_{A^{*}}\cdot P_{B^{*}};\;P_{B^{*}}=\frac{N_{B^{*}}}{{\mathrm{NS}}_{\mathrm{Ni},\mathrm{tot}}}\cdot Z^{m,m}$$

On the support, the reaction:

$$A^{v}+B^{v}↔{\mathrm{AB}}^{v}+v$$

has a forward rate expression, r_f_, given by:

rf=kf·NSs,tot·θAv.PBv;PBv=NBvNSs,tot·Zs,s

where for both equations, θ_X^a^_ is the fractional coverage of species X on site a ( $\theta_{A^{*}}=\frac{N_{A^{*}}}{{\mathrm{NS}}_{\mathrm{Ni},\mathrm{tot}}};\theta_{A^{v}}=\frac{N_{A^{v}}}{{\mathrm{NS}}_{s,\mathrm{tot}}}$ , for a = * and v, respectively) and P_B^a^_ is the probability of finding B^a^ next to A^a^. Z^m,m^ is the average surface coordination number on the Ni nanoparticle, calculated from a hemispherical Ni(111)-type surface model, wherein noninterfacial Ni atoms possess an in-plane CN of 6 and interfacial Ni atoms possess a CN of 4; the resulting Z^m,m^ varies depending on the nanoparticle size, with values approaching ∼6 for larger nanoparticle sizes.

3 $$Z^{m,m}=\frac{{\left(NS_{\mathrm{Ni},\mathrm{tot}}-NI_{\mathrm{Ni},\mathrm{tot}}\right)\times 6+NI_{\mathrm{Ni},\mathrm{tot}}\times 4}^{\;}}{NS_{\mathrm{Ni},\mathrm{tot}}}$$

Z^s,s^ is always set to a value of 3 based on the coordination number of surface O^v^ on a stoichiometric CeO_2_(111).

As noted earlier, the rates for noninterfacial reactions are a function of NS_Ni,tot_ or NS_s,tot_, depending on whether the reaction occurs on the metal or support. The site dependency is less straightforward for interfacial reactions that involve both metal and support sites. The rates are governed by NI_Ni,tot_ or NI_s,tot_, depending on the direction of the interfacial reaction. To provide further clarity, the forward rate expression for a H-spillover and the O-transport reaction are presented below.

For H-spillover, the reaction:

$$H^{*}+O^{v}↔OH^{v}+{\;}^{*}$$

has forward rate expression, r_f_:

$$r_{f}=k_{f}\cdot {\mathrm{NI}}_{\mathrm{Ni},\mathrm{tot}}\cdot \theta_{H^{*}}\cdot P_{O^{v}};P_{O^{v}}=\frac{N_{O^{v}}}{{\mathrm{NS}}_{s,\mathrm{tot}}}\cdot Z^{s,m}$$

For O-transport, the reaction:

$$O^{v}+*↔O^{*}+v$$

has forward rate expression, r_f_:

$$r_{f}=k_{f}\cdot {\mathrm{NI}}_{s,\mathrm{tot}}\cdot \theta_{O^{v}}\cdot P_{*};P_{*}=\frac{N_{*}}{{\mathrm{NS}}_{\mathrm{Ni},\mathrm{tot}}}\cdot Z^{m,s}$$

To keep the *K*_eq_ values constant, the reverse reaction rates for both H-spillover and O-transport reactions are set to be dependent on NI_Ni,tot_ and NI_s,tot_, respectively. Z^s,m^ is set to a constant value of 3, and Z^m,s^ is set to 2, in all analyses. Section 3.3 details the negligible effect of varying these parameters on the DRM rate/selectivity.

H-spillover rates are a function of the probability of finding a “O^v^” site neighboring the metal site (P_O^v^_). The dependency of forward and reverse H-spillover rate on NI_Ni,tot_, and not NI_s,tot_, allows the coverage of ‘OH^v^’ species to be higher than NI_s,tot_/NS_s,tot_. In other words, the “OH^v^” species access sites beyond the interfacial support region across the support, which is typical for reducible oxides.^64–66^

O-transport to the metal depends on the probability of finding an empty metal site (*) neighboring a support site (P_*_). The dependency of forward and reverse O-transport rate on NI_s,tot_ and not NI_Ni,tot_ restricts the concentration of O^v^ to be less than or equal to NI_s,tot_/NS_s,tot_, i.e., “O^v^” species that are only proximal to the metal particle are allowed to participate in O-transport.

Ordinary differential equations (ODE) are defined for 15 surface intermediates based on the formulated rate expressions. The system of ODE is solved for steady-state coverages using an in-house developed python code, with the “LSODA” numerical method in Python’s *solve_ivp* package. Steady-state coverage on metal and support converge to a sum of 2, with total coverage on metal and support individually summing up to 1. High numerical precision is ensured with absolute and relative tolerance parameters set to <10^–6^. The following section presents results and discussions, highlighting the model’s capability to elucidate interdependencies, provide mechanistic insights, and enable a holistic understanding of how functional parameters influence the overall DRM rate and selectivity.

## 3 Results and Discussion

### 3.1 Reaction Path Analysis

The MKM was first evaluated under a set of operating conditions (p_CH_4__ = p_CO_2__ = 0.5 bar; p_CO_ = p_H_2__ = p_H_2_O_ = 0; *T* = 973.15 K; *r*_m_ = 4 nm, *r*_int_ = 0.38 nm) to understand the reaction system and establish a reference for subsequent analyses of RDS as well as the effects of geometric and operational parameters.

The net steady-state rates are summarized in Figure 3 (forward and reverse rates and steady-state coverages of species included in the SI, Tables S11–S12). Reactions that are not part of the dominant pathway are represented with dashed arrows. The net DRM rate, which is equivalent to the net H_2_ production rate (r_R6_), equals 0.12 mol g_cat_^–1^ s^–1^. In contrast, the net rWGS rate is nearly negligible (r_R17_, 1.38 × 10^–6^ mol g_cat_^–1^ s^–1^) at these conditions; we note that the net rWGS rate is negligible for all conditions examined in this work and therefore will not be discussed in detail. As a result, selectivity, defined using the rate ratio, $\frac{r_{H_{2}}}{r_{\mathrm{CO}}}=\frac{r_{R6}}{r_{R10}+r_{R13}}$ , has a value of 0.99, nearly equal to the maximum theoretical value of unity. As necessitated by the reaction stoichiometry and the near-unity H_2_:CO ratio, the net CO production rates on the metal (r_R10_) and support (r_R13_) are equivalent, at 0.06 mol g_cat_^–1^ s^–1^. CH_4(g)_ and CO_2(g)_ adsorption reactions ([Disp-formula eq1]; 12 and 14; respectively) are quasi-equilibrated, with forward and reverse rates having large and comparable magnitudes (Table S11). Net rates for CH_4_* dehydrogenation and subsequent dehydrogenation ([Disp-formula eq2], 3, 4, and 5) have equivalent values of 0.06 mol g_cat_^–1^ s^–1^.

**Figure 3:**
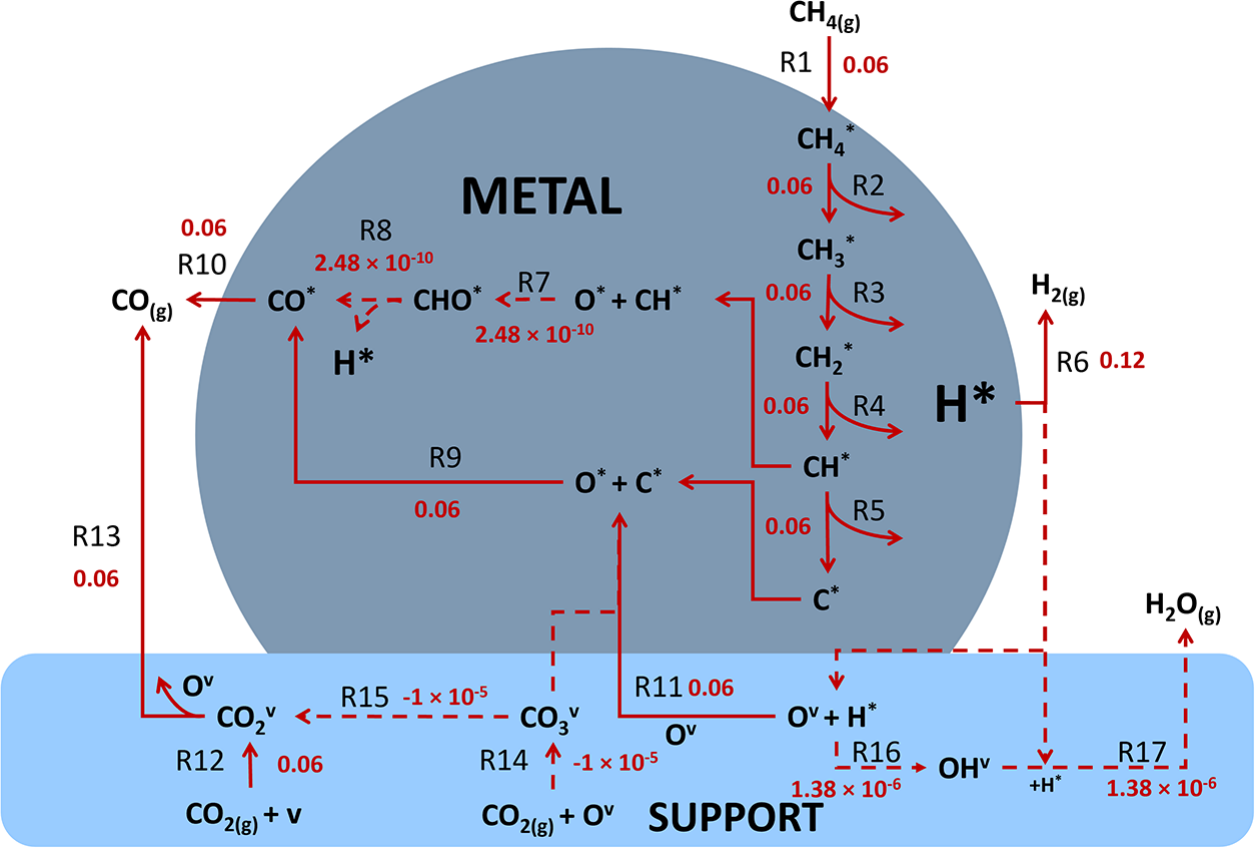
Net steady-state rates for all elementary reactions (in mol g_cat_^–1^ s^–1^), evaluated at p_CH_4__ = p_CO_2__ = 0.5 bar; p_CO_ = p_H_2__ = p_H_2_O_ = 0; *T* = 973.15 K; *r*_m_ = 4 nm; *r*_int_ = 0.38 nm. Bolded and dashed arrows represent reactions involved in dominant and minor pathways, respectively.

MKM allow examination of surface coverages for additional chemical insight (Table S12). Here, the Ni surface is nearly bare, with the main surface species being the C* (θ_C*_) ∼ 0.09 of a monolayer (ML). Oxidation of carbon species occurs primarily via the D1 pathway ([Disp-formula eq9]). As expected from the negligible surface coverage of CH*, the D2 pathway ([Disp-formula eq7], 8) contributes insignificantly to overall rates.

Among the interfacial reactions, net O-transport rates (r_R11_ + r_R15_; 0.06 mol g_cat_^–1^ s^–1^) are 4 orders of magnitude greater than net H-spillover rates (r_R16_ + r_R17_; 2.78 × 10^–6^ mol g_cat_^–1^ s^–1^). Despite the small H-spillover rates, the support is saturated with OH^v^ species (θ_OH^v^_) = 0.97, reflecting the large *K*_eq_ value (2.29 × 10^7^) of equilibrated [Disp-formula eq16], together with the large reaction barrier (2.4 eV) for water desorption ([Disp-formula eq17]).

#### 3.1.1 Rate or Selectivity-Controlling Kinetic Parameters

We identified the DRM rate-determining kinetic parameters using degree of rate control (DRC) analysis,^67^ which quantifies the sensitivity of overall rate (r_H_2__) to an infinitesimal change (1%) in the forward or reverse rate constant, while holding constant *K*_eq_ values. Similarly, selectivity-controlling parameters were identified using degree of selectivity control (DSC) analysis,^68^ defined as the sensitivity of product selectivity $\left(\frac{r_{H_{2}}}{r_{\mathrm{CO}}}\right)$ to an infinitesimal change (1%) in rate constants, with constant *K*_eq_ values. These results are presented in Figure 4 for the same reaction conditions as in the previous section.

**Figure 4:**
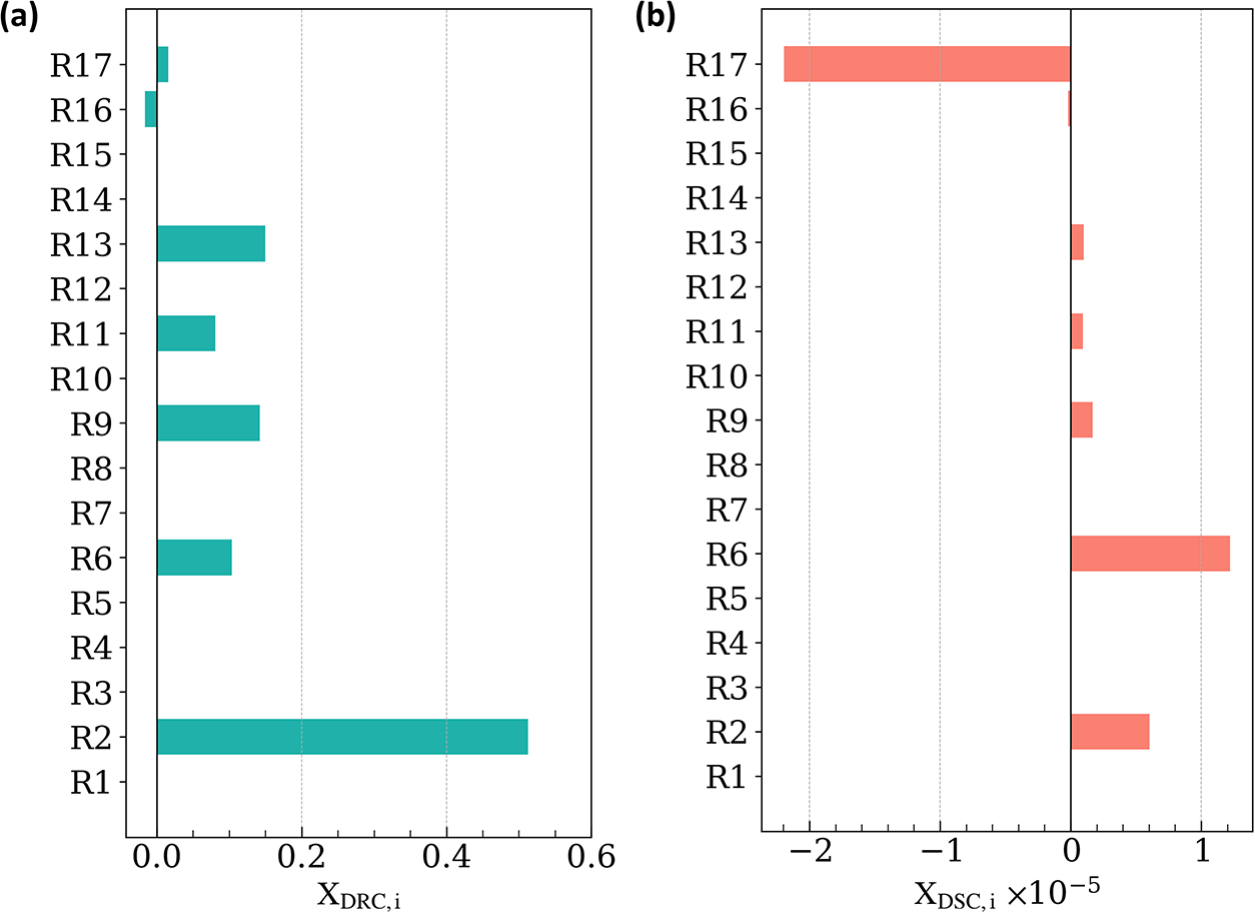
Degree of (a) rate and (b) selectivity control analysis at p_CH_4__ = p_CO_2__ = 0.5 bar; p_CO_ = p_H_2__ = p_H_2_O_ = 0; *T* = 973.15 K; *r*_int_ = 0.38 nm; *r*_m_ = 4 nm.

CH_4_* activation on Ni ([Disp-formula eq2]) is identified as the elementary step with the largest X_DRC,*i*_ value (0.51; Figure 4a) at these conditions. This is consistent with DRM studies reporting a strong dependence of rate on CH_4_ pressure.^69–72^ Four reactions, 6, 9, 11, and 13, also show positive yet small values of X_DRC,*i*_, meaning that accelerating these steps would enhance the overall rate. Promoting [Disp-formula eq9] suppresses θ_C*_ by generating bare metal sites for CH_4_ activation and increases the overall rate. Promoting [Disp-formula eq13] increases θ_O^v^_ and enables O-transport to Ni via [Disp-formula eq11], which in turn benefits [Disp-formula eq9] as evidenced by the equivalent net rates of [Disp-formula eq9] and 11 (Figure 3). These interactions demonstrate that [Disp-formula eq13] and 9 are coupled through 11, underscoring the role of interfacial O-transport in enhancing the overall rate. X_DRC,*i*_ of all other reactions contribute to ∼0.1% of the total X_DRC,*i*_ (∑_*i* = 1_^17^ X_DRC,*i*_ = 1), and thereby do not exert any control over the overall rate. All of the equilibrated reactions have negligible X_DRC,*i*_ values.

In contrast to the constraint by which the sum of the X_DRC*,i*_ values must be equal to unity, the sum of the X_DSC,*i*_ values is not constrained and can vary with reaction conditions. At these operating conditions, X_DSC,*i*_ values for all reactions are very small (∼10^–5^), consistent with H_2_-to-CO rate ratios that are high (∼0.99) and near the theoretical maximum (1). Among all reactions, 6 (associative H_2(g)_ desorption) and 17 (H_2_O_(g)_ formation by H-spillover) have relatively large positive and negative values, respectively (Figure 4b), indicating that H-spillover from the metal to the support plays a detrimental role in selectivity. However, these findings are based on 0% reactant conversion conditions. To accurately capture the H-spillover behavior, product cofeeding studies and/or integration of the MKM into a continuous reactor model are necessary.

### 3.2 Reactant Pressures and Their Effects on DRM Rate and Selectivity

Operational parameters often strongly influence the overall rate/selectivity. In this section, we examine the dependence of reaction rates on reactant (CH_4_ and CO_2_) partial pressures at 973.15 K.

#### 3.2.1 Influence of CH_4_ Pressure on DRM Rate

With p_CO_2__ fixed at 0.5 bar, the overall rate was evaluated over a wide range of p_CH_4__ (0.1–2.0 bar) for 4 nm Ni nanoparticles. Net DRM rate increases nearly linearly with an increase in CH_4_ pressure from 0.1 to 0.5 bar (Figure 5a). Correspondingly, the reaction order in CH_4_ in this regime is 0.89 (regression shown in Figure S1a), in agreement with experimentally reported values (0.9–1.0).^69–72^ As CH_4(g)_ pressure increases further (>0.7 bar), the DRM rate reaches a maximum value and then decreases with increasing CH_4(g)_ pressure, with reaction orders approaching values of −1.33 (regression shown in Figure S1b). This shift in the kinetic regime can be explained by the trends observed in θ_*_ and θ_C*_ (Figure 5b).

**Figure 5:**
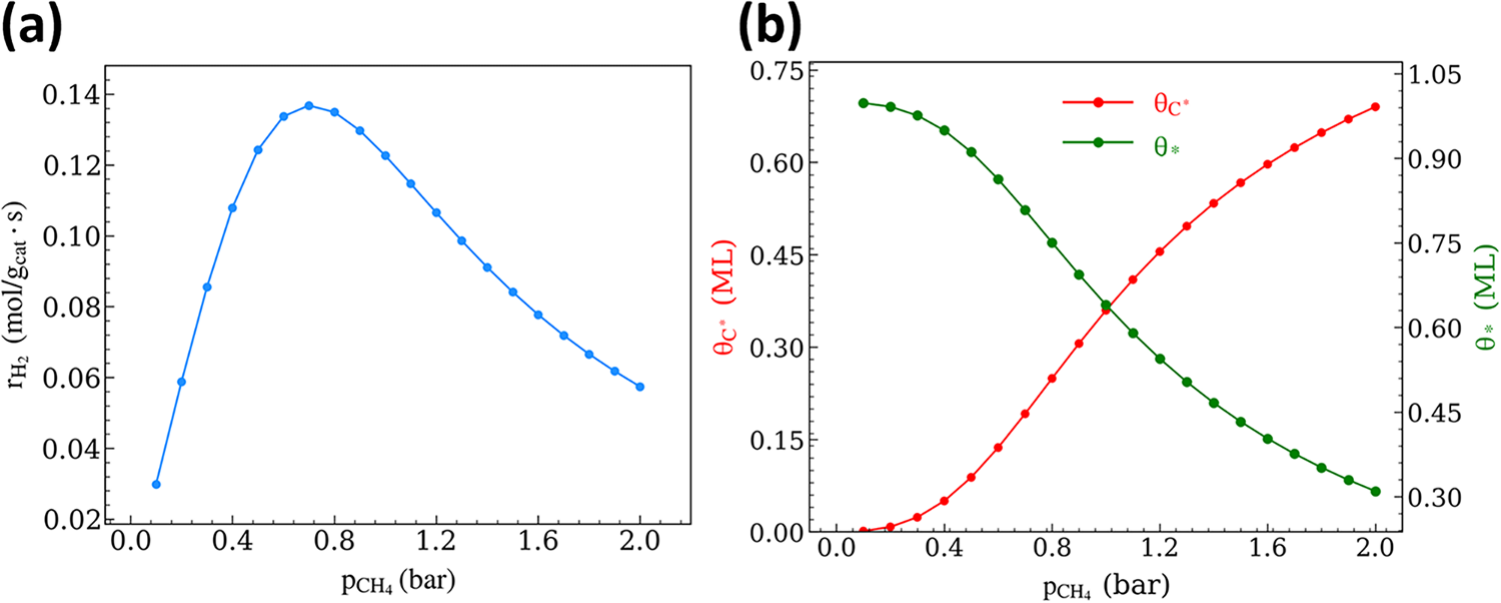
(a) DRM rate as a function of CH_4_ pressure and (b) steady-state coverages of Ni sites with C* and uncovered Ni sites, * (p_CO_2__ = 0.5 bar; p_CO_ = p_H_2__ = p_H_2_O_ = 0; *T* = 973.15 K; *r*_int_ = 0.38 nm; *r*_m_ = 4 nm).

In the positive reaction order regime (p_CH_4__ < 0.7 bar), the fraction of bare metal sites is high (θ_*_ > 0.8), with θ_C*_ < 0.2. However, with further increases in p_CH_4__, the coverage of Ni sites with C* also increases, with θ_C*_ exceeding 0.6 for p_CH_4__ > 1.5 bar. This behavior is accompanied by decreasing θ_*_ values (0.8 to 0.3 for p_CH_4__ from 0.7 to 2 bar). The normalized O* coverage (θ_O*_), already modest (0.01) at lower p_CH_4__ (Figure S2), decreases by nearly a factor of 30 at higher p_CH_4__ (>0.7 bar). Such a decrease in θ_O*_, together with an observed increase in θ_C*_, reflects interfacial O-transport rates that are insufficient to scavenge C* and form product CO, resulting in net DRM rates that decrease by nearly 15% (reaction path analysis shown in Figure S3). This behavior is also confirmed from Figure S4, where an increase in the ratio of forward rates of C* formation to O-transport (r_C*formation,f_/r_O‑transport,f_) is observed at higher p_CH_4__.

The mechanistic link between declining θ_O*_ and the overall rate is further validated by performing DRC analysis across the complete p_CH_4__ range. At lower p_CH_4__, 2 exerts the largest rate control (X_DRC,2_ ∼ 1), consistent with the measured reaction order (0.9). The negative shift in reaction order at higher p_CH_4__ (Figure 5a) is reflected by negative X_DRC,2_, accompanied by the increasing X_DRC,11_, approaching values of ∼1.5 (Figure 6a). In other words, 11, an interfacial O-transport reaction, becomes the RDS in this regime. Such trends, taken together with high values of θ_C*_, further affirm that O-transport is insufficient to oxidize the C* originating from CH_4_. Operating in such regions, where X_DRC,2_ is negative, would likely result in the formation of coke.

**Figure 6:**
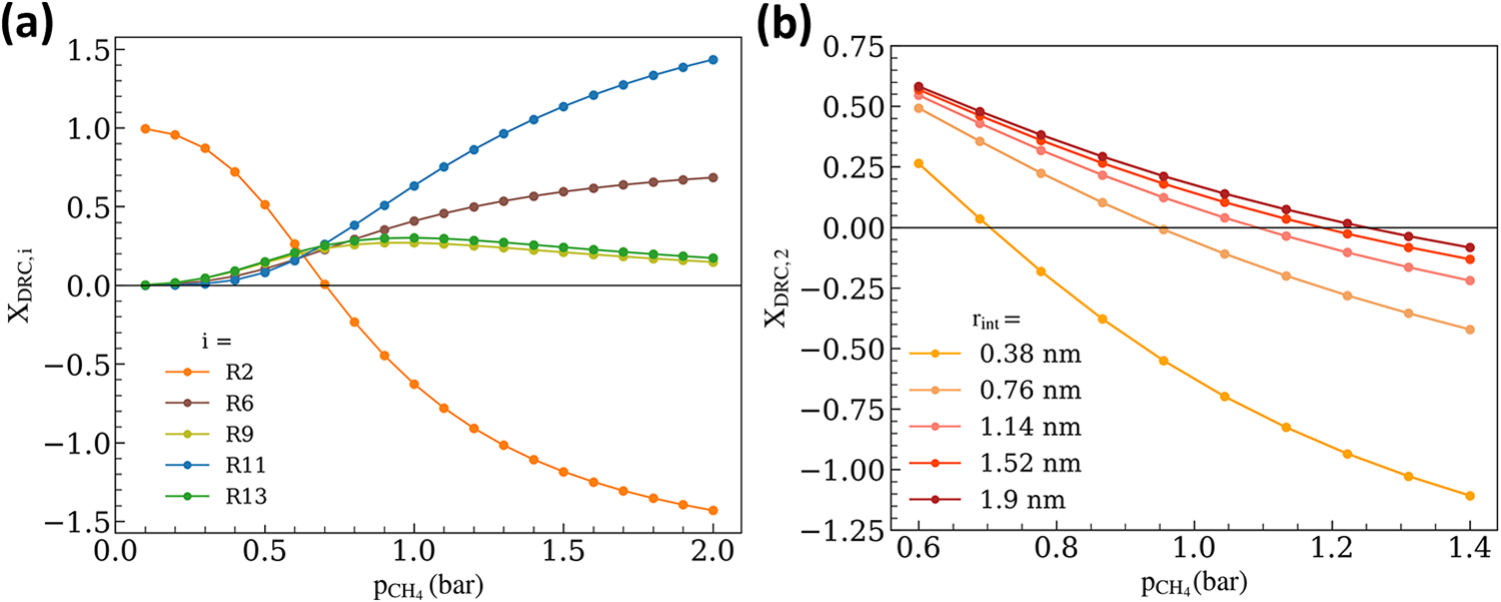
(a) DRC analysis as a function of p_CH_4__ for *r*_int_ = 0.38 nm, and (b) X_DRC,2_ values at varied *r*_int_ as a function of p_CH_4__ (p_CH_4__ = 0.6–1.4 bar, p_CO_2__ = 0.5 bar, p_CO_ = p_H_2__ = p_H_2_O_ = 0; *T* = 973.15 K; *r*_m_ = 4 nm).

The model captures a regime reflective of catalyst deactivation via the formation of carbonaceous deposits despite the exclusion of explicit elementary reactions for C–C bond formation for coke formation. This prediction purely arises from the relative rates of C* formation and O-transport, which determine the catalyst deactivation behavior. This demonstrates the robustness of this model in capturing structure–activity relationships and makes it a valuable tool for probing unrealistic or experimentally challenging operating regimes without the risk of reactor fouling due to coke formation.

Indeed, the balance between interfacial O-transport rates and C* formation rates (from CH_4_) can be examined by increasing *r*_int_, the interfacial radius. The X_DRC*,i*_ profiles for [Disp-formula eq2] at different *r*_int_ values are overlaid for p_CH_4__ = 0.6 to 1.4 bar (Figure 6b), highlighting the beneficial role of the doughnut-shaped interfacial support region in preventing formation of carbonaceous deposits on the Ni surface. Upon including the second support O-coordination shell for the Ni nanoparticle in the interfacial region (*r*_int_ = 0.76 nm), the inflection point, where X_DRC,2_ values become 0, increases from p_CH_4__ ≈ 0.7 to 0.9 bar. The inflection point monotonically shifts to higher methane pressures as *r*_int_ increases, although with decreasing sensitivity, specifically for extending the interfacial region beyond three support O-coordination shells (from *r*_int_ = 0.38 to 1.14 nm). Beyond such *r*_int_ values, the additional lattice O atoms do not contribute significantly to mitigating catalyst deactivation.

#### 3.2.2 Influence of CO_2_ Pressure on DRM Rate

Some DRM studies report a strong dependence of DRM rate on p_CO_2__ (reaction order ≥ 1).^69,71,73^ Here, the dependence was found to be weak for 0.1 ≤ p_CO_2__ ≤ 2.0 bar (Figure 7a), with a regressed reaction order of 0.24 (Figure S5c), in agreement with other studies that predict weaker dependency (reaction orders varying from 0 to 0.25). However, leveraging the advantage of an MKM to explore rate dependences at conditions that would experimentally be challenging, we sweep through narrow low-pressure ranges of CO_2_ (0.001–0.01 and 0.01–0.05 bar) and observe CO_2_ reaction orders of 0.72 and 0.52 (Figure S5a,b). Reaction orders in CO_2_ approach 1 at sufficiently low p_CO_2__ but become smaller at high p_CO_2__ (Figure 7a). Similar trends were reported on Ni–La_2_O_3_ and Ni–CeO_2_ catalysts,^70,72,74–76^ for which CH_4_ consumption rates exhibited strong dependences for lower p_CO_2__ (<5 kPa and <3 kPa, respectively), and near-zero dependences for higher p_CO_2__ values.

**Figure 7:**
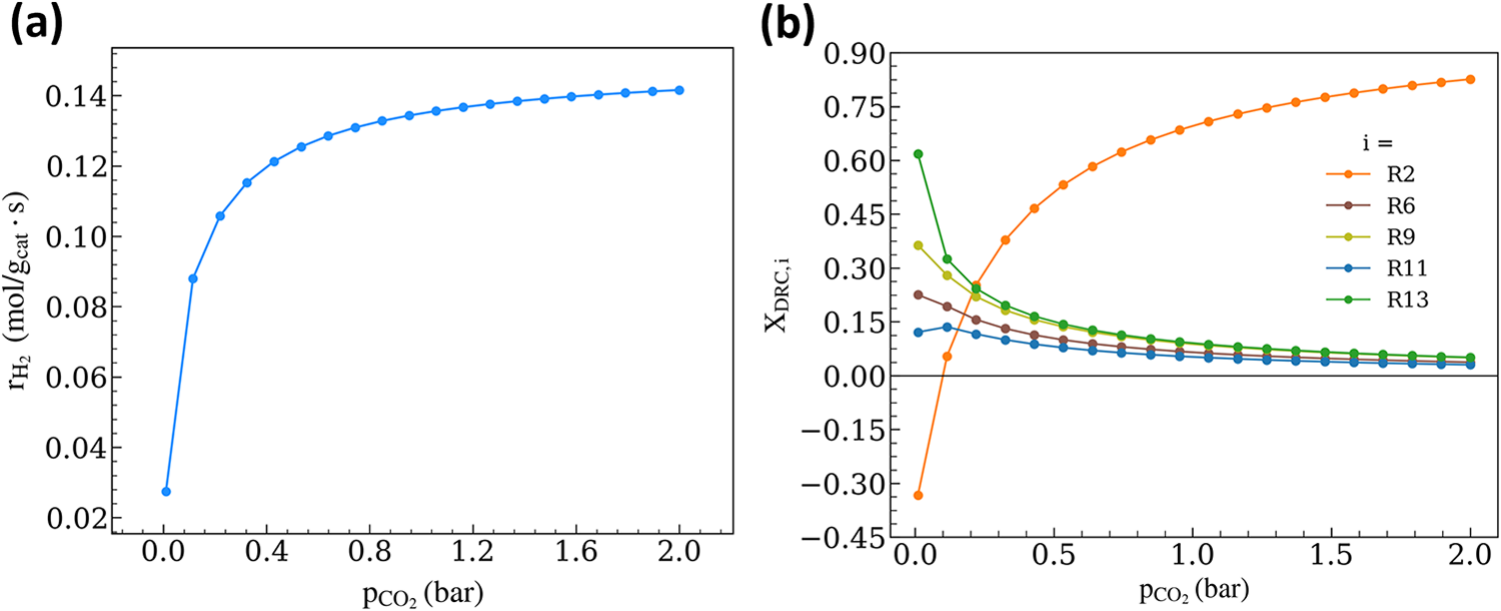
(a) DRM rate and (b) DRC analysis as a function of p_CO_2__ (p_CH_4__ = 0.5 bar; p_CO_ = p_H_2__ = p_H_2_O_ = 0; *T* = 973.15 K; *r*_int_ = 0.38 nm; *r*_m_ = 4 nm).

We perform DRC analysis for the complete p_CO_2__ region (Figure 7b), and observe negative X_DRC,2_ for p_CO_2__ < 0.09 bar (Figure S6a). As discussed earlier, this suggests a deactivation-prone regime resulting in coking (low values of θ_O*_, Figure S6b, and 0.28 ≤ θ_C*_ ≤ 0.58, Figure S6c). Similar to the scenario described in Section 3.2.1, where insufficient O-transport limits C* oxidation at high p_CH_4__ (Figure S4), the r_C*formation,f_/r_O‑transport,f_ ratio remains high at low p_CO_2__ (Figure S7), in agreement with the conclusions from rate and DRC analyses.

Nakamura et al. and Horiuichi et al. reported similar behavior on Rh/SiO_2_ and Ni/Al_2_O_3_ catalysts,^73,76^ where a reaction order of −0.6 and −0.27 was observed in CH_4_ while measuring a +1.0 and +0.21 order in CO_2_, respectively. These observations suggest a possible explanation for why DRM reaction orders are still debated. Variations across studies are attributable to differences in catalyst geometry, reaction conditions, and the partial pressure ranges investigated rather than inconsistencies in experimental procedures or modeling approaches. Section 3.4 provides insights into how similar variations can also arise from changes in metal particle size.

Despite the sensitivity of reaction rates to the concentration of each reactant (CH_4_ and CO_2_), the overall selectivity remains nearly constant at 0.99 (Figure S8), consistent with the negligible contribution of rWGS under the studied conditions. This sustained high selectivity may also be attributed to the omission of deactivation pathways, which prevents selectivity losses that typically arise from C_(s)_ formation via C* coupling.

### 3.3 Sobol Analysis for the Influence of Geometric Parameters on DRM Rate

Analyses in the preceding sections were conducted under largely constant geometric parameters. Figure 6b demonstrates variation in X_DRC,2_ values at different r_int_ values, indicating the potential role of geometric parameters in controlling the overall rate and/or selectivity. Because DRC represents a one-at-a-time sensitivity analysis formulated exclusively for kinetic parameters, it cannot capture sensitivities to other model parameters. Moreover, cross-parameter interactions may bias individual DRC analyses, yielding an incomplete understanding of the reaction system. To address these limitations, we employ a global sensitivity analysis method, specifically the Sobol method.

Sobol analysis was used to compute global sensitivities of other geometric parameters, along with the kinetic parameters to the overall reaction rate and selectivity.^70,75,77–79^ Given a user-defined range for each parameter and a sample size *N*, a sample space was generated based on the Saltelli sampling^80^ method. The MKM was solved for every point in the sample space, and the resulting rate data were utilized to calculate expectation and total variance defined as

4 $$V\left(Y\right)=\sum_{i=1}^{d}V_{i}+\sum_{i=1}^{d}\sum_{j>1}^{d}V_{\mathrm{ij}}$$

where *V_i_* and *V_ij_* are the first-order and second-order variances, respectively, calculated from the following expressions

5 $$V_{i}=V_{X_{i}}\left(E_{X_{∼i}}\left(Y|X_{i}\right)\right)$$

6 $$V_{\mathrm{ij}}=V_{X_{i},X_{j}}\left(E_{X_{∼i},j}\left(Y|X_{i},X_{j}\right)\right)-V_{i}-V_{j}$$

The two variances were used to calculate the first- and second-order sensitivity indices

7 $$S_{i}=\frac{V_{i}}{V\left(Y\right)}$$

8 $$S_{T,i}=\frac{V_{i}+V_{\mathrm{ij}}}{V\left(Y\right)}$$

These sensitivity indices, respectively, indicate isolated sensitivity of a parameter and sensitivity due to cross-parameter interactions. Sobol analysis was performed for 16 parameters (Table 5) with a sample size *N* of 9216; the value of *N* was selected based on the convergence of the sensitivity indices (Section S3, Figure S9). Sensitivity indices were assessed for both the rate and selectivity. All geometric parameters were varied over their full physical ranges to capture their true sensitivity. Partial pressures of reactants were varied by ±25% of the mean value of 0.5 bar while maintaining the reactant stoichiometry (1:1 p_CH_4__:p_CO_2__). The total pressure of the products was limited to 10% of the total reactant pressure. Parameter space chosen for the Sobol analysis is given in Table 5.

**Table 5:** Parameter Space for Sobol Sensitivity Analysis

parameter	range
*r*_m_ (nm)	2–10
*r*_int_ (nm)	0.25–1
Z^s,m^, Z^m,s^	1–4
p_CH_4__ = p_CO_2__ (bar)	0.375–0.625
p_H_2__ (bar)	0.03–0.05
p_CO_ (bar)	0.03–0.05
p_H_2_O_ (bar)	0.015–0.025
*k*_i,j_ (for [Disp-formula eq2], 6, 9, 11, 13, 15, 16, 17) (s^–1^)	±0.25 × *k*_*i*,*j*_

The radius of Ni nanoparticles (*r*_m_) yields the greatest values of the first- and second-order indices (Figure 8), suggesting that the DRM rates are most sensitive to changes in this geometric parameter. A notable difference between the first- and second-order sensitivities suggests strong cross-parameter interactions, meaning different behaviors for the same reaction system can be observed not only depending on the chosen particle size, but also based on other model parameters. The interactions are also present for overall selectivity analysis (Section S3, Figure S10), along with other parameters (*k*_6_, *k*_16_, Z^s,m^) exerting larger influence. However, analyses pertaining to their influence on rate or selectivity will not be discussed as a near-constant selectivity (∼0.99) is observed throughout the study.

**Figure 8:**
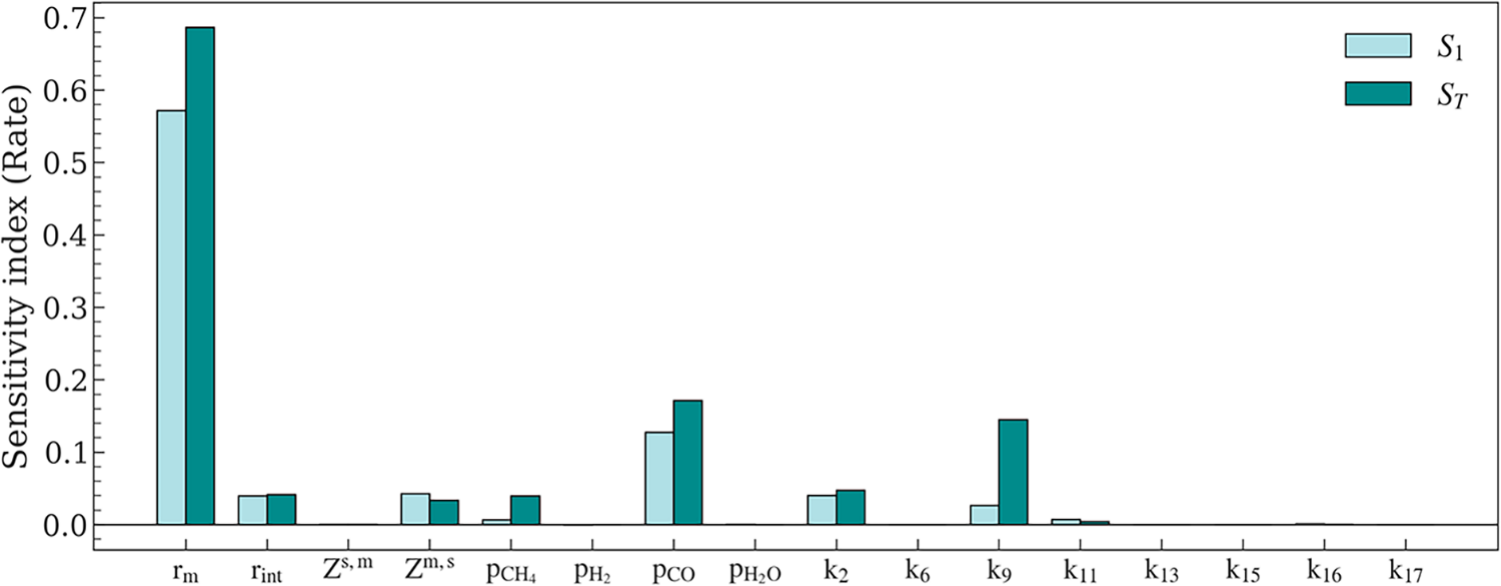
First- and second-order Sobol sensitivity indices, *S*_1_ and *S*_*T*_, respectively, of model parameters for DRM rate.

### 3.4 Influence of Ni Nanoparticle Size (*r*_m_) on DRM Rate

Before analyzing how variations in Ni nanoparticle size (*r*_m_) affect the RDS, it is essential to understand how individual reaction rates evolve with *r*_m_. For this, we perform a site evolution analysis as a function of *r*_m_, while maintaining a constant total metal-to-support active site ratio. For the particle size range considered here (2–10 nm), we assume that the activation barriers and reaction energies on metal and support sites remain constant and that size effects arise primarily from changes in the relative abundance of interfacial versus noninterfacial sites. Although reaction energetics may vary with particle size, particularly for subnanometer clusters due to significant changes in metal–metal coordination and metal–support interaction effects, explicitly capturing such behavior would require atomistic models of particle restructuring that are beyond the scope of the present study.

As r_m_ increases from 2 to 10 nm, the number of Ni nanoparticles decreases from ∼10^17^ to 10^15^ due to particle agglomeration, consistent with the expected decreases in surface area-to-volume ratios of nanoparticles of increasing size (schematic representation shown in the SI, Figure S11a,b). Consequently, the metal dispersion decreases from 37 to 7% (Figure S11c), while the ratio of exposed support sites (NS_s,tot_/NS_Ni,tot_) increases from 7 to 36 (Figure 9a). The increase in metal nanoparticle size also decreases the fraction of support sites which are interfacial $\left(\frac{NI_{s,\mathrm{tot}}}{NS_{s,\mathrm{tot}}}\right)$ , as shown in Figure 9b, reflecting fewer Ni nanoparticles with a constant interfacial radius, *r*_int_.

**Figure 9:**
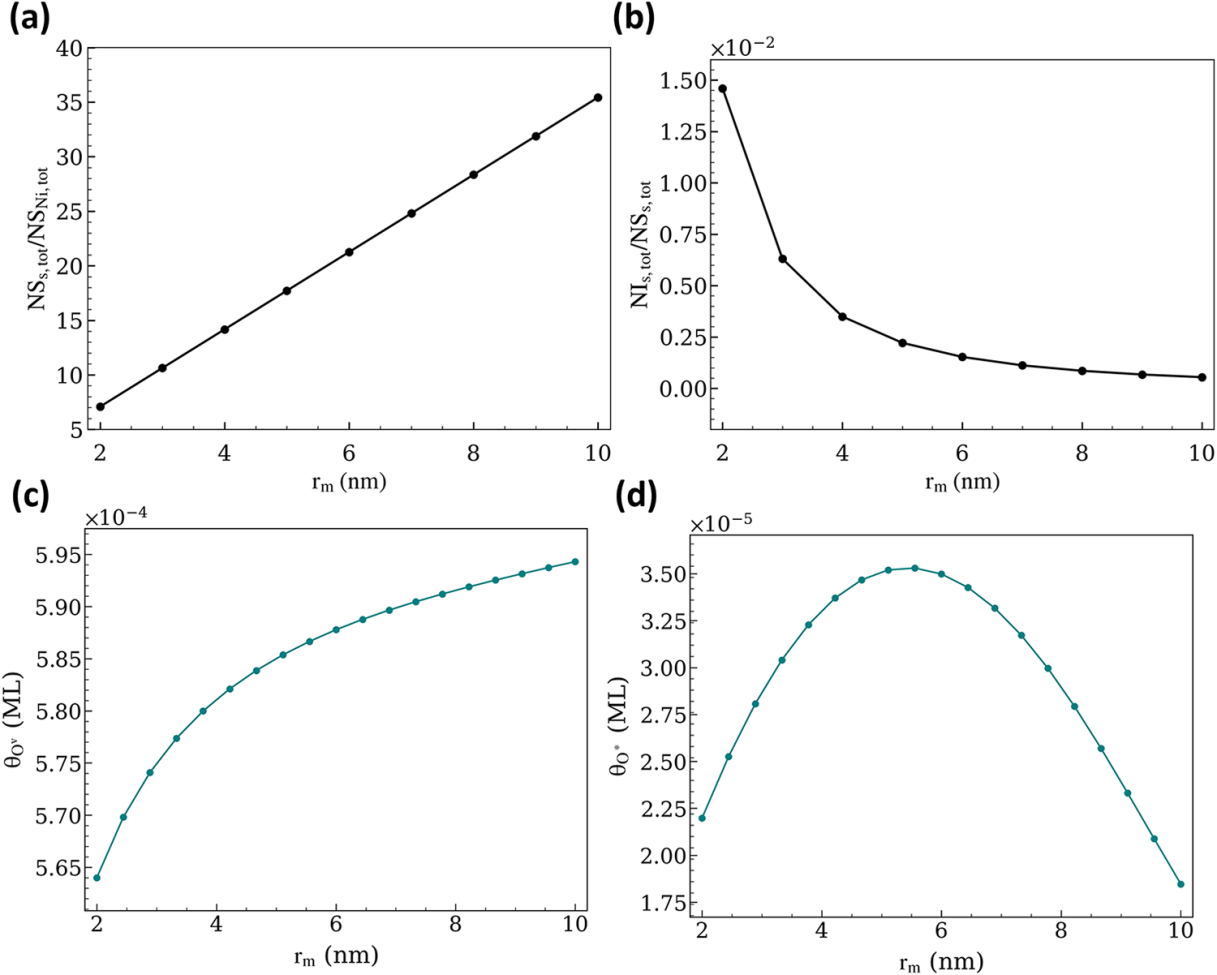
(a, b) Site evolution analysis for varying nanoparticle size. (c, d) Steady-state coverage of O^v^ and O* species as a function of *r*_m_ at p_CH_4__ = p_CO_2__ = 0.5 bar; p_CO_ = p_H_2__ = p_H_2_O_ = 0; *T* = 973.15 K; *r*_int_ = 0.38 nm.

The decrease in NS_Ni,net_ at a larger r_m_ causes the overall H_2_ and CO production rates (r_R6_ and r_R10_) to decrease from ∼10^–1^ to ∼10^–2^ mol g_cat_^–1^ s^–1^ (Figure 10a,b). Although higher NS_s,tot_/NS_Ni,tot_ might intuitively favor CO production on the support ([Disp-formula eq13]), this effect is offset by the decreasing net interfacial reaction rates (Figure 10c,d). At these conditions, the coverage of O^v^ species increases on the support (Figure 9c). Interestingly, the coverage of O* exhibits a volcano-shaped dependence on *r*_m_ (Figure 9d). This trend arises from the competing effects of decreasing fraction of interfacial sites, NI_s,tot_/NS_s,tot_ (Figure 9b) and increasing the O^v^ coverage on the support (Figure 9c). At small to intermediate Ni nanoparticle sizes, the increase in θ_O^v^_ contributes to an increase in θ_O*_, until a maximum is reached at *r*_m_ = 5 nm. Beyond this size, however, the relative abundance of interfacial sites decreases sharply, limiting the rate of O-transport, and thereby the O* coverage on the metal. Consequently, θ_C*_ increases in this large-particle regime (Figure S12a).

**Figure 10:**
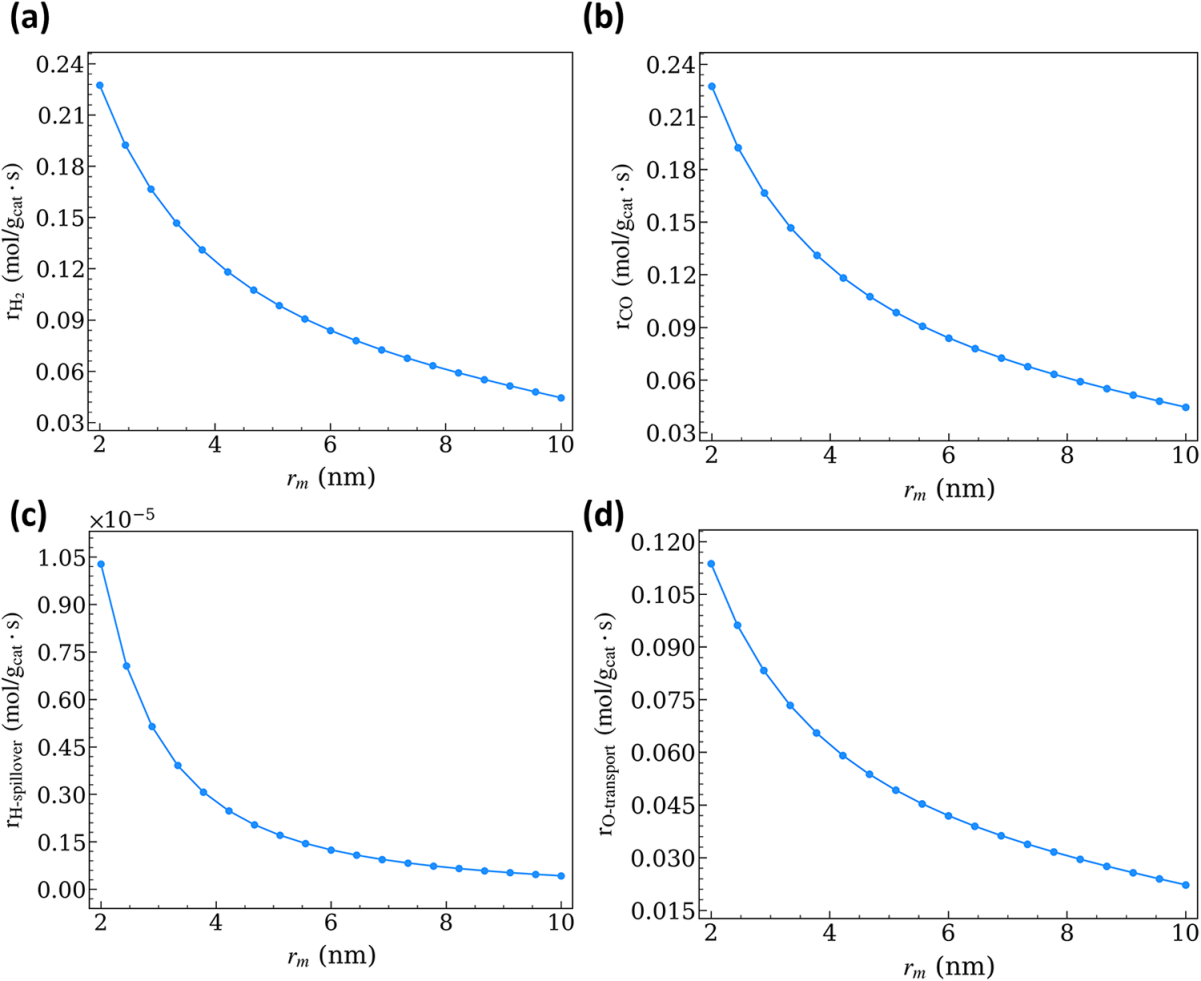
Net rate of (a) H_2_ formation, (b) CO formation, (c) H-spillover, and (d) O-transport as a function of *r*_m_ (p_CH_4__ = p_CO_2__ = 0.5 bar; p_CO_ = p_H_2__ = p_H_2_O_ = 0; *T* = 973.15 K; *r*_int_ = 0.38 nm).

To examine the effect of this behavior on the RDS, DRC analysis was performed at particle sizes ranging from 2 to 10 nm. As shown in Figure 11, 2 has the highest X_DRC,*i*_ value (∼0.5) at a smaller *r*_m_ (<5 nm). This control diminishes as *r*_m_ increases, with an inflection point at *r*_m_ = 8.4 nm. The r_C*formation,f_/r_O‑transport,f_ behavior (Figure S13) is similar to that observed in Section 3.2.1; one would expect a higher C* coverage at these conditions, along with the negative X_DRC,2_ values and increasing dominance of [Disp-formula eq11] (X_DRC,11_ ∼ 0.8 for *r*_m_ = 10 nm). Though θ_C*_ increases for particles larger than 5 nm, the model predicts a θ_C*_ of only 0.11 ML for a 10 nm particle, which is comparable to that observed for a 2 nm particle (θ_C*_, θ_OH*_, θ_*_, and θ_v_ included in the SI, Figure S12). Thus, the negative rate control of [Disp-formula eq2] is not driven by excessive C* coverage but rather by the substantial drop in the O-transport rate at *r*_m_ = 10 nm relative to *r*_m_ = 2 nm (Figure 10d). Reaction pathway analyses on small versus larger Ni nanoparticles are provided in Figure S14 to provide a complete overview of the reaction progression. The decline in O-transport rate despite similar θ_O*_ across the sampled particle sizes (Figures 9d, S14) is attributed to the rapid decrease in NI_s,tot_ and NS_Ni,tot_ for larger particles. Thus, this example illustrates that a deactivation regime does not always coincide with high θ_C*_, and such a dependence on NI_s,tot_ also means that widening the interfacial doughnut region will shift the deactivation regime farther, similar to the observations presented in Figure 6b. We note that despite these differences, selectivity increases negligibly with increasing *r*_m_ values (as shown in Figure S15).

**Figure 11:**
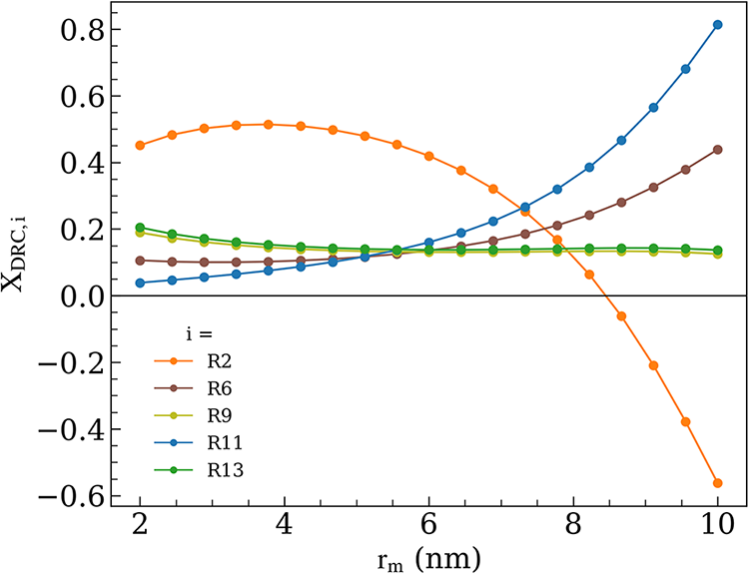
DRC analysis as a function of *r*_m_ (p_CH_4__ = p_CO_2__ = 0.5 bar; p_CO_ = p_H_2__ = p_H_2_O_ = 0; *T* = 973.15 K; *r*_int_ = 0.38 nm).

### 3.5 Potential Future Research Directions

Despite the model’s ability to predict catalyst deactivation behavior and reproduce experimental reaction orders, the interfacial effects captured here remain lumped due to certain simplifying approximations. Several research extensions can naturally build upon this framework as an advancement.

In particular, the current framework does not explicitly assign distinct energetics to interfacial sites. While the DFT-derived kinetic parameters used in this study already embed key metal–support electronic interactions, an explicit treatment of the interface with unique interfacial sites, species, and elementary reactions would allow decoupling of the currently lumped interfacial effects and enable a deeper mechanistic understanding of interfacial dynamics. Second, incorporating facet-dependent kinetics, for example, accounting for different facets of CeO_2_ and estimating their respective surface chemistries, may refine quantitative predictions. Stabilization strategies such as molecular sieve confinement or oxide core–shell architectures are commonly employed to maintain Ni dispersion at high temperatures. Incorporating such effects into this model will enable a more realistic description of interfacial dynamics.

Additionally, cofeeding experiments, such as introducing H_2_ or H_2_O with reactants, could help probe interfacial H-spillover pathways and their effects on product distributions. Our preliminary H_2_ cofeeding test (1% H_2_) indicates a strong inhibitory effect: steady-state C* coverage increases by an order of magnitude (from 0.09 to 0.50) as we cofeed 1% H_2_, suggesting the prevalence of coke formation. The r_C*formation,f_/r_O‑transport,f_ ratio at this condition is ∼20,000, compared to 3.06 in the absence of cofeed. The DRC analysis is consistent with the observations, as R11 (O-transport reaction) becomes the rate-determining step with X_DRC,11_ ∼1. We surmise that this behavior is the result of higher H-spillover rates, which consume lattice O^v^ on the support thereby hindering O-transport rates to the metal. Collectively, the significant sensitivities of surface coverages and DRC values to gas-phase conditions motivate the use of an integrated reactor model to decode the inherent complexity of the DRM system.

## 4 Conclusions

A multisite MKM framework was developed to elucidate interfacial species transport effects, using the DRM + rWGS system as a case study. A metal–support site quantification approach enabled precise control of the interfacial region, allowing modulation of interfacial reaction rates and their effect on the overall rate. Sobol analysis identified the metal particle radius, *r*_m_, as a globally influential geometric parameter.

CH_4_ activation remains the sole RDS at lower p_CH_4__, or for smaller Ni particles, whereas O-transport becomes rate-limiting at higher CH_4_ concentration or larger metal particles, suggesting the prevalence of catalyst deactivation. Under these conditions, CH_4_ activation exerts a negative influence on the overall rate. A wider interfacial “doughnut” region can potentially shift this deactivation regime. The model’s ability to predict deactivation-prone regimes without explicit coking mechanisms, using only a multisite framework, enables exploration of experimentally inaccessible conditions, providing comprehensive theory-guided catalyst testing. The variation in CO_2_ reaction order across pressure regimes, showing near-zero order at high pressures and significantly higher order at low pressures, is in agreement with experimental reports, thereby demonstrating the model’s capability to capture distinct operational regimes. Overall, this framework offers a versatile and predictive approach for investigating interfacial phenomena in catalytic systems, paving the way for rational catalyst design through tailored metal–support interactions.

## Supplementary Material



## Data Availability

DFT structures and MKM codes used in this work are available in this GitHub repository: https://github.com/shenoy1099/DRM-MKM.git.

## Notes

Additional model development details, including detailed active site quantification and kinetic parameter estimation and adjustment using Sequential Least Squares Programming; steady-state net rates, surface coverages, reaction path analyses, and other model results and analyses, at additional values of model parameters; and Sobol analysis results (PDF)
