# Supplementary material for: A Multisite Microkinetic Framework for Describing Interfacial Kinetics in Dry Methane Reforming (DRM) over Ni-CeO2 Catalysts
Source: ACS Catal. 2026 Jan 14;16(3):2535–49. doi: 10.1021/acscatal.5c07743 (PMC12887931; doi:10.1021/acscatal.5c07743)
Supplement: Supplementary file 1 [file cs5c07743_si_001.pdf]

Supporting Information

for

A Multi-Site Microkinetic Framework for Describing Interfacial Kinetics in Dry Methane Reforming (DRM) over Ni-CeO$_2$ Catalysts

Nirenjan Shenoy Padmanabha Naveen[a], Kerry M. Dooley[b], Michael J. Janik[a], Gina Noh[a,*], Konstantinos Alexopoulos[a,*]

[a] Department of Chemical Engineering, The Pennsylvania State University, University Park, Pennsylvania, 16802, United States

[b] Department of Chemical Engineering, Louisiana State University, Baton Rouge, Louisiana, 70803, United States

[*]kxa5325@psu.edu, gnoh@psu.edu

Section S1. Active site quantification from catalyst model

This section contains detailed active site calculations for both the metal (Ni nanoparticles) and support ($CeO_2(111)$), with information on their respective interfacial site quantification.

S1A. Ni metal site quantification

Below is an example calculation to demonstrate the steps followed for quantifying metal sites for Ni nanoparticles (r_m = 2nm) deposited on $CeO_2(111)$

- Assume:
  i. Total catalyst loading = 1g
  ii. Total Ni loading[1] = 4 wt%
  iii. r_m = 2 nm
- Total mass of Ni, $m_{Ni} = 0.04$ g
- Total moles of Ni, $n_{Ni} = \frac{0.04\ \mathrm{g}}{58.69\ \mathrm{gmol}} = 6.8 * 10^{-4}$ mol
- Total Ni sites in 0.04g Ni, $N_{Ni} = 6.022 * 10^{23} * 6.8 * 10^{-4} = 4.10 * 10^{20}$
- Volume occupied by 1 Ni particle, $V_p = \frac{2}{3} * \Pi * r_m^3 = \frac{2\Pi}{3} * 8\ \mathrm{nm}^3$
- Radius occupied by 1 Ni atom in the fcc bulk structure, $r_{fcc} = \frac{a}{2\sqrt{2}} = \frac{0.35}{2\sqrt{2}} = 0.124$ nm
  Lattice constant for the bulk fcc Ni was set as a = 0.35 nm [2]
- Volume occupied by 1 fcc Ni atom, $V_a = \frac{4\pi}{3} * (0.124)^3\ \mathrm{nm}^3$
- Total Ni atoms in 1 Ni particle, $N_{Ni,p} = \frac{V_p}{V_a} = 2098$
- Curved surface area of 1 Ni particle, $CSA_{Ni,p} = 8\pi\ \mathrm{nm}^2$
- Volume of the outermost shell of the hemispherical particle, $V_{shell} = CSA_{Ni,p} * (2*r_{fcc}) = 6.23$ nm^3
- Total surface Ni atoms in 1 Ni particle, $NS_{Ni,p} = V_{shell}/V_a = \frac{6.2329}{\frac{4\pi}{3}*(0.124)^3} = 780.44$
- Ni dispersion = $\frac{NS_{Ni,p}}{N_{Ni,p} - NS_{Ni,p}} * 100 = \frac{780.4370}{2098 - 780.4370} * 100 = 59.23\%$
- Circumference of 1 Ni particle = $2 * \pi * r_m = 4\pi$ nm
- Total interfacial metal sites in 1 Ni particle, $NI_{Ni,p} = \frac{\text{Circumference}}{\text{diameter of 1 Ni fcc atom}} = \frac{4\pi}{0.248} = 50.67$
- Total Ni particles in 4wt% Ni, $N_p = \frac{N_{Ni}}{N_{Ni,p}} = \frac{4.1*10^{20}}{2098} = 1.95 * 10^{17}$
- Total interfacial sites $NI_{Ni,tot} = NI_{Ni,p} * N_p = 9.90*10^{18}$
- Total surface metal sites ($NS_{Ni,tot}$) = $NS_{Ni,p} * N_p = 1.53*10^{20}$
- Total projected surface area occupied by 4wt% Ni, $A_m = N_p$ * Area occupied by 1 Ni particle $= 1.9542 * 10^{17} * \pi * (2 * 10^{-9})^2 = 2.46\ \mathrm{m}^2$

S1B. CeO_2(111) support site quantification

The total support-based active sites ($NS_{s,tot}$) is calculated from the exposed surface area of the support for a given projected surface area of 4 wt% Ni (A_m). The calculations below are based on 2 nm Ni particles deposited CeO_2(111).

- Assume:
  i. Specific surface area of catalyst[1] - 70 $m^2\ g^{-1}$

  ii. Interfacial support radius, r_{int} = 0.25 nm

- Total projected surface area occupied by CeO_2(111), $A_s = 70 - A_m = 67.54\ m^2\ g^{-1}$
- Areal density of lattice O^v on CeO_2 (111):
  Using DFT calculations in this study, we determine the areal density of O^v (coordinatively saturated and unsaturated) to be 15.45 nm^{-2} as we consider both surface and sub-surface O^v to be active for any elementary reaction.
- Total O^v sites available in 67.54 m^2/g ($NS_{s,tot}$) = $\frac{15.45}{10^{-18}} * 67.544 = 1.04 * 10^{21}$
- Number of interfacial support sites around a single Ni nanoparticle ($NI_{s,p}$) = $4\pi * (r_S^2 - r_m^2) *$ areal O^vdensity $= 1.06\pi * 15.45 = 51.42$
- Total number of interfacial sites ($NI_{s,tot}$) = $NI_{s,p} * N_p = 51.42 * 1.95 * 10^{17} = 1.00 * 10^{19}$

Section S2. Estimation and adjustment of kinetic parameters in the microkinetic model

S2A. Literature references for kinetic/thermodynamic parameters

This section contains a collection of activation enthalpies ($\Delta \mathbf{H}_\mathbf{f}^\ddagger$) and reaction enthalpies ($\Delta \mathbf{H}_\mathbf{rxn}$) for some important elementary reactions (*R2, R6, R13*) considered in this study, obtained from different DFT studies on Ni_n/CeO_2 (or) Ni(111) catalyst models. This information is provided to show the magnitude of variation in kinetic/thermodynamic parameters.

R2 - CH_4^* activation step

Table S1. DFT estimated activation and reaction enthalpies for reaction R2.

**Catalyst model**	$\Delta \mathbf{H}_\mathbf{f}^\ddagger \left(\frac{\mathbf{kJ}}{\mathbf{mol}}\right)$	$\Delta \mathbf{H}_\mathbf{rxn} \left(\frac{\mathbf{kJ}}{\mathbf{mol}}\right)$	**Reference**
Ni_1-CeO_2(111)	84.92	8.685	[3]
	83.955	-1.93	[4]
Ni_3-CeO_2(111)	113.87	-0.965	[4]
Ni_4-CeO_2(111)	13.51	-77.2	[4]
	94.57	5.79	[5]
	80.095	4.825	[6]
	98.43	-6.755	[7]
	98.43	-1.93	[8]
Ni_6-CeO_2(111)	8.685	-51.145	[4]
Ni_8-CeO_2(111)	41.495	-2.895	[9]
	86.85	41.495	[9]
Ni_{10}-CeO_2(111)	80.095	4.825	[10]
Ni_{13}-CeO_2(111)	32.81	30.88	[4]
	34.74	1.93	[4]
Ni(111)	86.85	-8.685	[4]
	75.27	-15.44	[6]
	87.815	86.85	[11]

R6 - Associative H^* desorption step

Table S2. DFT estimated activation and reaction enthalpies for reaction R6.

**Catalyst model**	$\Delta H_f^{\ddagger}\left(\frac{kJ}{mol}\right)$	$\Delta H_{rxn}\left(\frac{kJ}{mol}\right)$	**Reference**
Ni_1-$CeO_2(111)$	85.89	85.89	12
Ni_2-$CeO_2(111)$	195.90	195.90	12
Ni_4-$CeO_2(111)$	128.35	128.35	13
Ni_8-$CeO_2(111)$	128.35	128.35	14
Ni_{10}-$CeO_2(111)$	183.35	183.35	15
Ni_{13}-$CeO_2(111)$	74.31	74.31	16
Ni(111)	109.05	109.05	14
	82.99	-57.90	17
	110.01	5.79	11

R13 - CO_2 activation on support step

Table S3. DFT estimated activation and reaction enthalpies for reaction R13.

**Catalyst model**	$\Delta H_f^{\ddagger}\left(\frac{kJ}{mol}\right)$	$\Delta H_{rxn}\left(\frac{kJ}{mol}\right)$	**Reference**
$CeO_2(111)$	164.05	138.96	18
	126.42	81.06	19
$CeO_2(110)$	134.14	127.38	20
	259.59	238.36	20
	155.37	102.29	21

S2B. Adjustment of kinetic parameters for thermodynamic consistency

This section presents the process of adjusting kinetic parameters for achieving thermodynamic consistency. Kinetic parameters for both forward and backward reactions ($\Delta H_i^{\ddagger}$ and $\Delta S_i^{\ddagger}$) are initially obtained from existing DFT literature (**Table S6**). Overall reaction enthalpies and entropies for individual reaction pathways are estimated (**Table S7**), and compared to NIST gas phase data (**Table S5**). Total error in the overall ΔH_{rxn} and ΔS_{rxn} is minimized by adding modest corrections to the kinetic parameters using a structured methodology (**Table S9, S10**). All activation and reaction enthalpy values are shown in kJ/mol; entropy values usually have much lower order of magnitudes relative to enthalpies, thus, are represented in J mol^{-1} K^{-1}.

Gas phase thermochemical data for DRM and rWGS reactions

Table S4 displays the thermochemical data of gas phase species at 973.15 K. These were obtained from the NIST-JANAF[22] thermochemical tables via linear interpolation between 900 and 1000 K. Using those values, and calculated ΔS_f^0 for each species, we compute the reaction energies and entropies, as given in the table below. Because the analyses carried out in this work are at 973.15 K, we calculate the free energy at this single temperature. These values will be used as the reference for evaluating thermodynamic consistency of individual reaction paths.

Table S4. Enthalpy of formation (ΔH_f^0), and Gibbs free energy (ΔG_f^0) for gas phase species at 0.1 MPa and 973.15 K

Species	$\Delta H_f^0 \left(\frac{kJ}{mol}\right)$	$\Delta G_f^0 \left(\frac{kJ}{mol}\right)$
$CH_{4(g)}$	-89.54	16.57
$CO_{2(g)}$	-394.56	-395.85
$H_{2(g)}$	0.00	0.00
$H_2O_{(g)}$	-247.68	-194.06
$CO_{(g)}$	-111.83	-197.90

Table S5. Overall stoichiometric reaction enthalpies (ΔH_{rxn}) and entropies (ΔS_{rxn}) for DRM/rWGS reactions

Reaction	$\Delta H_{rxn} \left(\frac{kJ}{mol}\right)$	$\Delta S_{rxn} \left(\frac{J}{mol.K}\right)$	$\Delta G_{rxn} \left(\frac{kJ}{mol}\right)$ @ 700 °C
DRM<br>$CH_{4\,(g)} + CO_{2\,(g)} \leftrightarrow 2CO_{(g)} + 2H_{2\,(g)}$	260.44	284.60	-16.52
rWGS<br>$CO_{2\,(g)} + H_{2\,(g)} \leftrightarrow CO_{(g)} + H_2O_{(g)}$	35.05	32.03	3.89

Table S6. Unmodified kinetic parameters obtained from DFT literature

Rxn #	Reaction	$\Delta H_f^{\ddagger}$ (kJ /mol)	$\Delta H_r^{\ddagger}$ (kJ /mol)	$\Delta H_{rxn,i}$ (kJ /mol)	A_f (s^{-1})	A_r (s^{-1})	$\Delta S_{rxn,i}$ (J/mol K)	Ref [¥]
R1	$CH_{4(g)} + * \leftrightarrow CH_4^*$	0.00	1.93	-1.93	3.47E+08	2.06E+14	-112.45	10
R2	$CH_4^* + * \rightarrow CH_3^* + H^*$	**94.56**	**88.77**	**5.79**	6.75E+11	6.08E+13	-37.41	10 5
R3	$CH_3^* + * \rightarrow CH_2^* + H^*$	74.30	30.88	43.42	1.26E+13	1.56E+13	-1.77	10
R4	$CH_2^* + * \rightarrow CH^* + H^*$	50.17	63.68	-13.51	6.68E+12	2.31E+13	-10.32	10
R5	$CH^* + * \leftrightarrow C^* + H^*$	67.54	81.05	-13.51	1.49E+13	1.36E+13	0.79	10
R6	$2H^* \leftrightarrow H_{2(g)} + 2*$	74.30	0.00	74.30	**2.14E+13**	9.99E+07	**102.00**	23 **16** 24
R7	$CH^* + O^* \leftrightarrow CHO^* + *$	147.63	104.21	43.42	1.51E+13	6.66E+12	6.78	10 11
R8	$CHO^* + * \leftrightarrow CO^* + H^*$	19.30	142.81	-123.51	2.54E+13	1.62E+13	3.75	10 11
R9	$C^* + O^* \leftrightarrow CO^* + *$	107.10	130.26	-23.16	1.32E+13	1.47E+12	18.26	10
R10	$CO^* \leftrightarrow CO_{(g)} + *$	**68.51**	**0.00**	**68.51**	3.26E+19	2.62E+12	135.76	10
R11	$O^v + * \leftrightarrow O^* + v$	133.16	136.05	-2.89	1.77E+13	3.46E+18	-101.25	10 25
R12	$CO_{2(g)} + v \leftrightarrow CO_2^v$	0.00	1.93	-1.93	3.47E+08	2.61E+14	-112.45	23
R13	$CO_2^v \leftrightarrow CO_{(g)} + O^v$	**167.89**	**75.26**	**92.63**	**2.92E+13**	**2.21E+13**	2.31	
R14	$CO_{2(g)} + O^v \leftrightarrow CO_3^v$	**17.37**	**50.17**	**-32.81**	**3.28E+11**	**1.29E+13**	-30.51	
R15	$CO_3^v + * \leftrightarrow CO_2^v + O^*$	171.75	70.73	101.03	2.03E+13	2.03E+10	57.41	
R16	$H^* + O^v \leftrightarrow OH^v + *$	**119.65**	**234.47**	**-114.82**	**1.81E+13**	**3.44E+13**	-5.34	
R17	$OH^v + H^* \leftrightarrow H_2O_{(g)} + v + *$	**218.63**	**194.85**	**23.78**	**1.61E+14**	**1.78E+13**	18.32	

[¥] *References are color-coded for better visual correlation; bolded black fonts are used for values estimated via in-house DFT calculations*

Using the above kinetic parameters (**Table S6**) does not satisfy thermodynamic consistency; the table below indicates the error between DFT-calculated and the NIST values of ΔH_{rxn} and ΔS_{rxn} for each DRM and rWGS pathway, along with error estimation for the entropic term at 700 °C.

Table S7. Error calculations for reaction enthalpy/entropy for DRM/rWGS pathways

	**D1**	**D2**	**W1**	**W2**
**$\Delta H_{rxn,calculated}$ (kJ/mol)**	302.01	258.59	-74.64	-1.31
**$\Delta S_{rxn,calculated}$ (J/mol.K)**	-14.52	-23.04	-199.16	41.43
**Error in ΔH_{rxn} (kJ/mol)**	41.50	-1.92	-109.42	-36.09
**Error in ΔS_{rxn} (J/mol.K)**	41.57	-1.85	-109.69	-36.65
**Error in $T\Delta S_{rxn}$ (kJ/mol)**	-299.12	-307.65	-231.19	9.41

The acceptable error margin in ΔH_{rxn} and $T\Delta S_{rxn}$ is set at $\pm$ 10 kJ mol^{-1} in this work. Errors in ΔH_{rxn} closely satisfy this margin for all pathways except W1. Entropic terms produce errors that are ~2 orders of magnitude higher than the margin, suggesting huge corrections to be applied on ΔS based terms. Inconsistency is also noted for linearly dependent reaction combinations in the model. One such example is: R14 + R15 – R11 = R12; $\Delta H_{net}(\Delta H_{R14} + \Delta H_{R15} - \Delta H_{R11}) = \Delta H_{R12}$ and $\Delta S_{net}(\Delta S_{R14} + \Delta S_{R15} - \Delta S_{R11}) = \Delta S_{R12}$

	ΔH_{net} (kJ/mol)	ΔS_{net} (J/mol.K)	$T\Delta S_{net}$ (kJ/mol)
R14 + R15 – R11	161.14	29.21	28.43
R12	-1.93	-112.45	-109.43

Sequential Least Squares Programming method for correcting kinetic parameters

This is a constrained optimization method that provides the smallest total corrections to be applied for each kinetic parameter. A total of four equations are formulated individually for enthalpic- and entropic-based corrections. These equations impart overall thermodynamic consistency with gas-phase NIST data, and are subjected to a linear inequality problem with allowable errors in net ΔX_{DRM} or ΔX_{rWGS}.

**Sum of individual reaction X values (LHS) for a given pathway**	**RHS for X = H**	**RHS for X = S**
$\Delta X_1 + \Delta X_2 + \Delta X_3 + \Delta X_4 + \Delta X_5 + 2 * \Delta X_6 + \Delta X_9 + \Delta X_{10} + \Delta X_{11} + \Delta X_{12} + \Delta X_{13}$	260.44	284.60
$\Delta X_1 + \Delta X_2 + \Delta X_3 + \Delta X_4 + 2 * \Delta X_6 + \Delta X_7 + \Delta X_8 + \Delta X_9 + \Delta X_{10} + \Delta X_{11} + \Delta X_{12} + \Delta X_{13}$		
$\Delta X_{12} + \Delta X_{13} + \Delta X_{16} + \Delta X_{17} - \Delta X_6$	35.05	32.03
$\Delta X_{13} + \Delta X_{14} + \Delta X_{15} + \Delta X_{16} + \Delta X_{17} - \Delta X_6 - \Delta X_{11}$		

Two constraints are included while solving the algebraic inequalities: (1) The net ΔX_{DRM} or ΔX_{rWGS} may not exceed the NIST-obtained values by ± 9 J/mol.K (2) Corrections to any reaction enthalpy ($\Delta H_{rxn,\,Ri}$) may not exceed ± 25 kJ/mol (this number was chosen to satisfy the maximum error allowable in net ΔH_{DRM} or ΔH_{rWGS}); similarly, corrections to any reaction entropy ($\Delta S_{rxn,\,Ri}$) may not exceed ± 100 J/mol.K. More freedom for corrections on entropic terms is allowed due to the larger magnitude of errors associated with ΔS_{DRM} or ΔS_{rWGS} than the respective enthalpic terms (**Table S7**).

The individual terms carry weights (w_i) based on their hypothesized sensitivity towards overall rate (**Table S8**); this will minimize the corrections added to such sensitive reactions and compensate them over the kinetically unimportant reactions. Weights for all reactions were initially set to 1, but modified in an iterative way, so as to facilitate satisfying the two constraints associated with the algebraic inequalities.

Table S8. Weights associated with each elementary reaction enthalpy ($\Delta H_{rxn,i}$) and entropy ($\Delta S_{rxn,i}$), defined for solving the algebraic inequalities

Reaction i	Weight for $\Delta H_{rxn,i}$	Weight for $\Delta S_{rxn,i}$
R1	10	0.1
R2	1	1
R3	0.1	0.1
R4	0.1	0.1
R5	0.1	0.1
R6	1	0.1
R7	0.01	0.01
R8	0.01	0.01
R9	1	1
R10	0.1	0.1
R11	0.1	0.1
R12	10	0.1
R13	1	1
R14	0.1	1
R15	0.1	0.1
R16	0.1	0.1
R17	0.1	0.1

A quadratic objective function is defined: minimize $\delta X \sum_{i=1}^{n} w_i(\delta X_i)^2$, where δX_i is the correction term - $\Delta\Delta H_{rxn,i}$ or $\Delta\Delta S_{rxn,i}$. The SLSQP solver is called in Python to solve for the linear inequalities. The objective function is minimized using Lagrange multipliers that handle bounds and linear constraints, to find a global minimum. Since this is a quadratic objective function, we expect no local minimum, which also imparts more confidence in the corrected kinetic parameters. The corrections in $\Delta H_{rxn,i}$ and $\Delta S_{rxn,i}$ are presented in **Table S9** and **S10**.

Enthalpic corrections

Any corrections to individual $\Delta H_{rxn,\,Ri}$ are equally distributed between $\Delta H_f^{\ddagger}$ and $\Delta H_r^{\ddagger}$ for reactions having non-zero $\Delta H_f^{\ddagger}$ and $\Delta H_r^{\ddagger}$, and only to $\Delta H_f^{\ddagger}$ or $\Delta H_r^{\ddagger}$ where one of them is zero.

*For R1, -0.08 kJ/mol is distributed between $\Delta\Delta H_f^{\ddagger}$ and $\Delta\Delta H_r^{\ddagger}$ as 0.92 and 1.00 kJ/mol respectively.

#For R6, -25.00 kJ/mol is solely imparted to $\Delta\Delta H_f^{\ddagger}$, given $\Delta\Delta H_r^{\ddagger}$ =0.

Table S9. Corrections for individual reaction enthalpies, represented as $\Delta\Delta H_{rxn,i}$

**Rxn i**	**Reaction**	**$\Delta H_{rxn,i}$ (kJ/mol)**	**$\Delta\Delta H_{rxn,i}$ (kJ/mol)**
R1	$CH_{4(g)} + * \leftrightarrow CH_4^*$	-1.93	-0.08*
R2	$CH_4^* + * \rightarrow CH_3^* + H^*$	5.79	-0.75
R3	$CH_3^* + * \rightarrow CH_2^* + H^*$	43.42	-7.51
R4	$CH_2^* + * \rightarrow CH^* + H^*$	-13.51	-7.51
R5	$CH^* + * \leftrightarrow C^* + H^*$	-13.51	-8.32
R6	$2H^* \leftrightarrow H_{2(g)} + 2*$	74.30	-25.00#
R7	$CH^* + O^* \leftrightarrow CHO^* + *$	43.42	8.13
R8	$CHO^* + * \leftrightarrow CO^* + H^*$	-123.51	8.13
R9	$C^* + O^* \leftrightarrow CO^* + *$	-23.16	-0.83
R10	$CO^* \leftrightarrow CO_{(g)} + *$	68.51	-7.51
R11	$O^v + * \leftrightarrow O^* + v$	-2.89	24.24
R12	$CO_{2(g)} + v \leftrightarrow CO_2^v$	-1.93	2.62
R13	$CO_2^v \leftrightarrow CO_{(g)} + O^v$	92.63	23.07
R14	$CO_{2(g)} + O^v \leftrightarrow CO_3^v$	-32.81	-3.18
R15	$CO_3^v + * \leftrightarrow CO_2^v + O^*$	101.03	-25.00
R16	$H^* + O^v \leftrightarrow OH^v + *$	-114.82	25.00
R17	$OH^v + H^* \leftrightarrow H_2O_{(g)} + v + *$	23.78	25.00

Entropic corrections

Any corrections to individual $\Delta S_{rxn,i}$ is imparted by multiplying a factor α to the original A_f/A_r of the elementary reaction, where $\alpha = \exp\left(\frac{\Delta\Delta S_{rxn,i}}{R}\right)$; the overall entropic correction is distributed between forward and backward reaction pre-exponential factor terms ($A_{f,new} = A_{f,old} * \sqrt{\alpha}$; $A_{r,new} = \frac{A_{r,old}}{\sqrt{\alpha}}$).

Table S10. Corrections to individual reaction entropies, represented as $\Delta\Delta S_{rxn,i}$

**Rxn i**	**Reaction**	$\Delta S_{rxn,i}$ $\left(\frac{J}{mol\ K}\right)$	$\Delta\Delta S_{rxn,i}$ $\left(\frac{J}{mol\ K}\right)$
R1	$CH_{4(g)} + * \leftrightarrow CH_4^*$	-112.45	23.58
R2	$CH_4^* + * \rightarrow CH_3^* + H^*$	-37.41	2.36
R3	$CH_3^* + * \rightarrow CH_2^* + H^*$	-1.77	23.58
R4	$CH_2^* + * \rightarrow CH^* + H^*$	-10.32	23.58
R5	$CH^* + * \leftrightarrow C^* + H^*$	0.79	21.94
R6	$2H^* \leftrightarrow H_{2(g)} + 2*$	102.00	-6.71
R7	$CH^* + O^* \leftrightarrow CHO^* + *$	6.78	16.33
R8	$CHO^* + * \leftrightarrow CO^* + H^*$	3.75	16.33
R9	$C^* + O^* \leftrightarrow CO^* + *$	18.26	2.19
R10	$CO^* \leftrightarrow CO_{(g)} + *$	135.76	23.58
R11	$O^v + * \leftrightarrow O^* + v$	-101.25	75.02
R12	$CO_{2(g)} + v \leftrightarrow CO_2^v$	-112.45	100.00
R13	$CO_2^v \leftrightarrow CO_{(g)} + O^v$	2.31	7.74
R14	$CO_{2(g)} + O^v \leftrightarrow CO_3^v$	-30.51	-5.14
R15	$CO_3^v + * \leftrightarrow CO_2^v + O^*$	57.41	-51.44
R16	$H^* + O^v \leftrightarrow OH^v + *$	-5.34	53.87
R17	$OH^v + H^* \leftrightarrow H_2O_{(g)} + v + *$	18.32	53.87

Table S11. Steady state net rates for elementary reactions at $p_{CH_4} = p_{CO_2} = 0.5$ bar ; $p_{CO} = p_{H_2} = p_{H_2O} = 0$; T = 973.15 K ; interfacial radius (r_{int}) = 0.38 nm ; metal nanoparticle radius (r_m) = 4 nm

**Reaction i**	**r_f (mol g_{cat}^{-1} s^{-1})**	**r_r (mol g_{cat}^{-1} s^{-1})**	**net rate (mol g_{cat}^{-1} s^{-1})**
R1	7.39E+04	7.39E+04	6.21E-02
R2	6.21E-02	4.97E-07	6.21E-02
R3	6.21E-02	2.87E-06	6.21E-02
R4	6.21E-02	2.49E-06	6.21E-02
R5	5.23E-01	4.61E-01	6.20E-02
R6	1.24E-01	0.00E+00	1.24E-01
R7	2.48E-10	6.91E-16	2.48E-10
R8	2.48E-10	7.98E-17	2.48E-10
R9	6.21E-02	5.84E-13	6.21E-02
R10	6.21E-02	0.00E+00	6.21E-02
R11	1.71E-01	1.09E-01	6.20E-02
R12	3.37E+06	3.37E+06	6.21E-02
R13	6.21E-02	0.00E+00	6.21E-02
R14	1.78E+04	1.78E+04	-1.00E-05
R15	1.01E-05	2.01E-05	-1.00E-05
R16	1.92E-05	1.78E-05	1.38E-06
R17	1.38E-06	0.00E+00	1.38E-06

Table S12. Steady state coverage of all surface species at $p_{CH_4} = p_{CO_2} = 0.5$ bar ; $p_{CO} = p_{H_2} = p_{H_2O} = 0$; $T = 973.15$ K ; interfacial radius (r_{int}) = 0.38 nm ; metal nanoparticle radius (r_m) = 4 nm

Species	Coverage, ML
*	9.11×10^{-1}
CH_4^*	1.33×10^{-5}
CH_3^*	1.07×10^{-8}
CH_2^*	1.02×10^{-9}
CH^*	3.45×10^{-8}
C^*	8.86×10^{-2}
H^*	7.16×10^{-5}
O^*	3.30×10^{-5}
CO^*	6.86×10^{-15}
CHO^*	9.59×10^{-20}
CO_2^v	3.18×10^{-3}
CO_3^v	3.40×10^{-4}
OH^v	9.65×10^{-1}
O^v	5.81×10^{-4}
v	3.10×10^{-2}

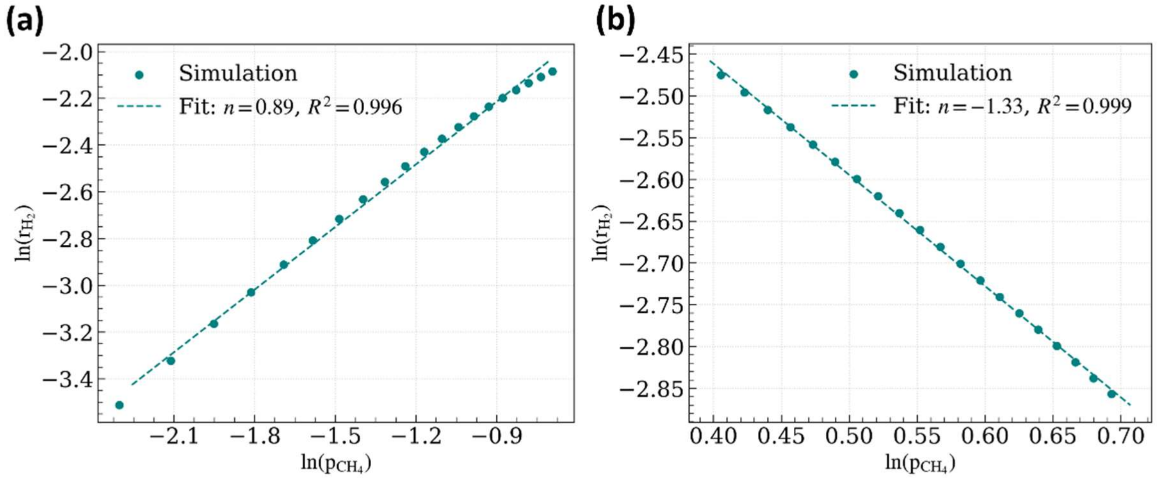

Figure S1. $\ln(r_{H_2})$ vs $\ln(p_{CH_4})$ fitting for CH_4 reaction orders at **(a)** p_{CH_4} = 0.1 to 0.5 bar or **(b)** p_{CH_4} = 1.5 to 2.0 bar. Other reaction conditions: $p_{CO_2} = 0.5$ bar; $p_{CO} = p_{H_2} = p_{H_2O} = 0$; $T = 973.15$ K ; $r_{int} = 0.38$ nm ; $r_m = 4$ nm

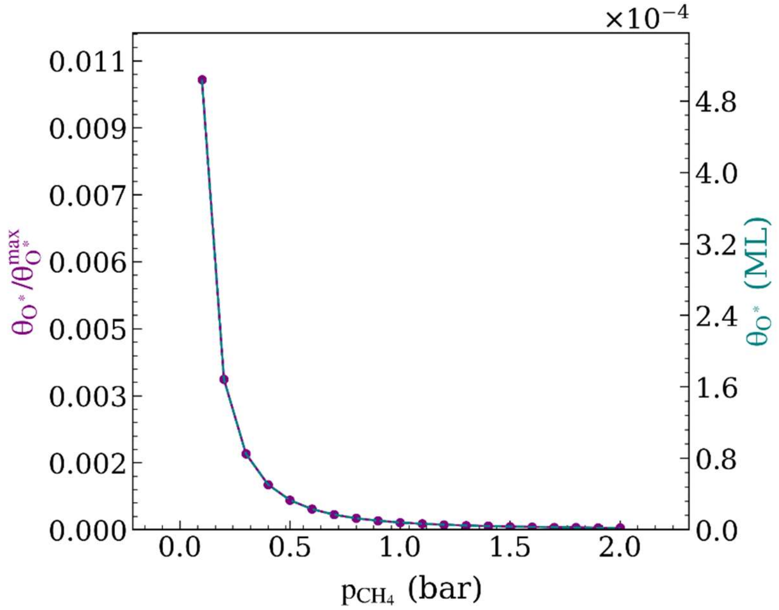

Figure S2. Steady state $\frac{\theta_{O^*}}{\theta_{O^*}^{max}}$, θ_{O^*} as a function of p_{CH_4} at $p_{CO_2} = 0.5$ bar; $p_{CO} = p_{H_2} = p_{H_2O} = 0$; $T = 973.15$ K ; $r_{int} = 0.38$ nm ; $r_m = 4$ nm

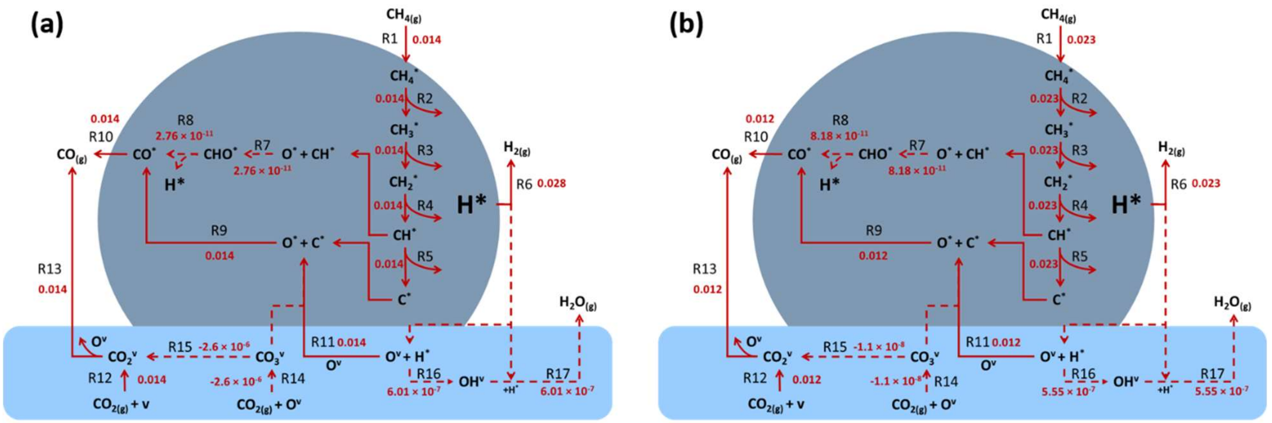

Figure S3. Reaction path analysis at **(a)** $p_{CH_4} = 0.1$ bar; $p_{CO_2} = 0.5$ bar **(b)** $p_{CH_4} = 2.0$ bar; $p_{CO_2} = 0.5$ bar. Other reaction conditions: $p_{CO} = p_{H_2} = p_{H_2O} = 0$; $T = 973.15$ K ; $r_{int} = 0.38$ nm ; $r_m = 4$ nm. Bolded and dashed arrows represent reactions that are part of the dominant and minor reaction pathways, respectively. All rates are expressed in mol g_{cat}^{-1} s^{-1}

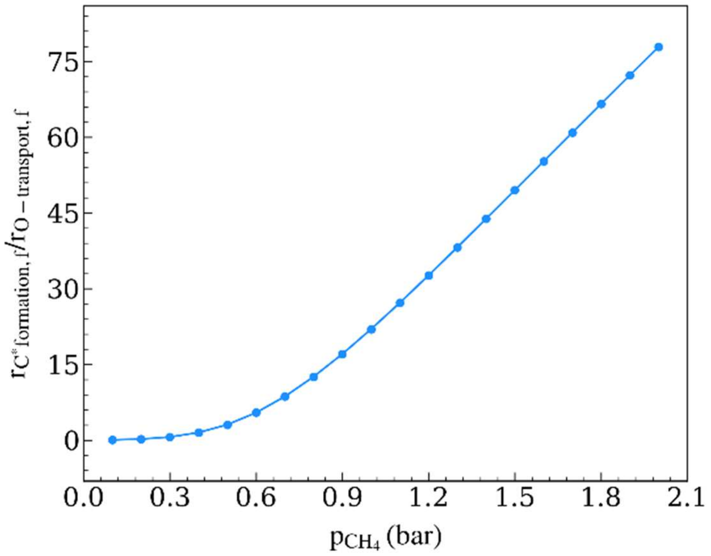

Figure S4. Ratio of forward rates of C^* formation to O-transport ($r_{C^*formation,f}/r_{O\text{-}transport,f}$) as a function of p_{CH_4} at $p_{CO_2} = 0.5$ bar; $p_{CO} = p_{H_2} = p_{H_2O} = 0$; $T = 973.15$ K ; $r_{int} = 0.38$ nm ; $r_m = 4$ nm

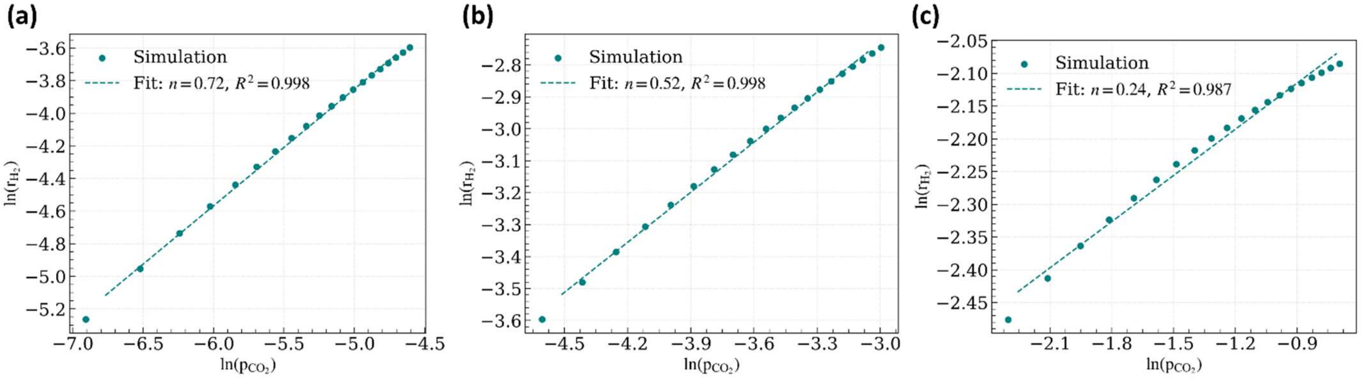

Figure S5. $\ln(r_{H_2})$ vs $\ln(p_{CO_2})$ analysis for **(a)** p_{CO_2} = 0.001 to 0.01 bar **(b)** p_{CO_2} = 0.01 to 0.05 bar **(c)** p_{CO_2} = 0.1 to 0.5 bar at $p_{CH_4} = 0.5$ bar ; $p_{CO} = p_{H_2} = p_{H_2O} = 0$; T = 973.15 K ; $r_{int} = 0.38$ nm ; $r_m = 4$ nm

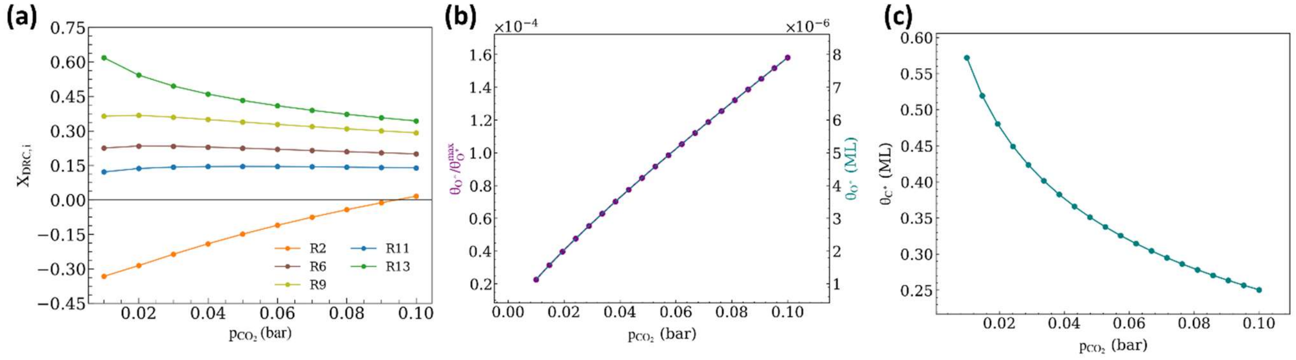

Figure S6. (a) DRC analysis, **(b)** Steady state $\frac{\theta_{O^*}}{\theta_{O^*}^{max}}$, θ_{O^*}, **(c)** Steady state θ_{C^*} plots as a function of p_{CO_2} = 0.01 to 0.1 bar, at $p_{CH_4} = 0.5$ bar ; $p_{CO} = p_{H_2} = p_{H_2O} = 0$; T = 973.15 K ; $r_{int} = 0.38$ nm ; $r_m = 4$ nm

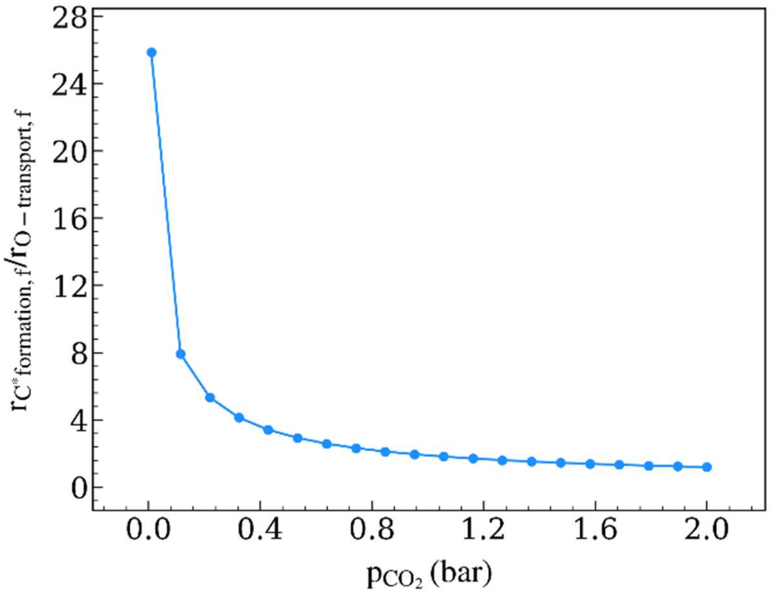

Figure S7. Ratio of forward rates of C^* formation to O-transport ($r_{C^*formation,f}/r_{O\text{-}transport,f}$) as a function of p_{CO_2} at $p_{CH_4} = 0.5$ bar; $p_{CO} = p_{H_2} = p_{H_2O} = 0$; $T = 973.15$ K ; $r_{int} = 0.38$ nm ; $r_m = 4$ nm

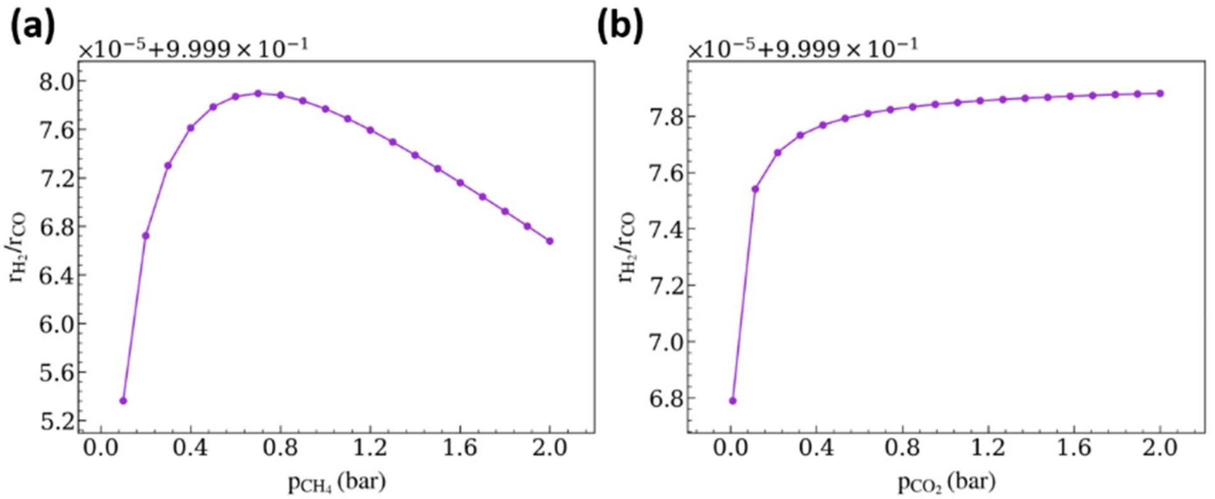

Figure S8. Selectivity as a function of **(a)** p_{CH_4} (0.1 to 2.0 bar) and **(b)** p_{CO_2} (0.01 to 2.0 bar) at $p_{CO} = p_{H_2} = p_{H_2O} = 0$; $T = 973.15$ K ; $r_{int} = 0.38$ nm ; $r_m = 4$ nm

Section S3. Sobol analysis

The shaded regions around each curve represent the 95% CI region. Despite the relatively wide confidence-interval regions at any sample size, we observe the top three parameters to appear consistently at any sample size.

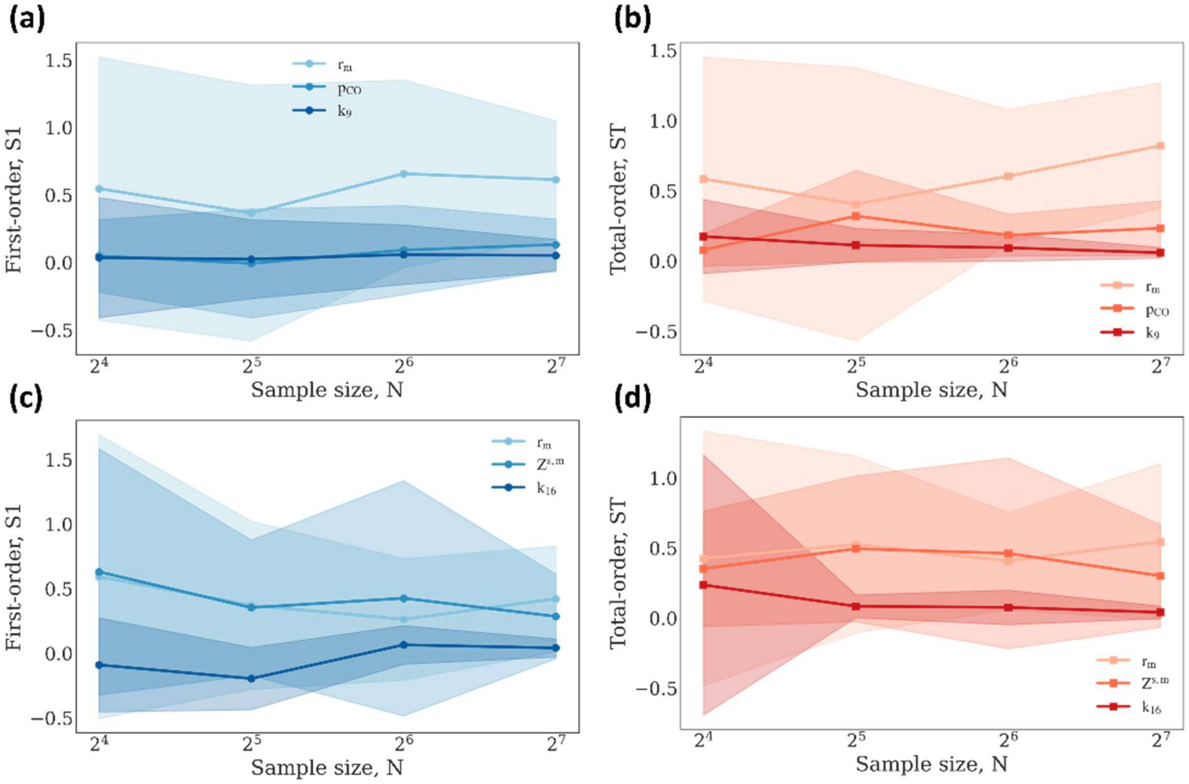

Figure S9. Convergence of Sobol sensitivity indices (first and second order, S_1 and S_T) with sample size (N) for **(a)-(b)** DRM rate, and **(c)-(d)** DRM selectivity

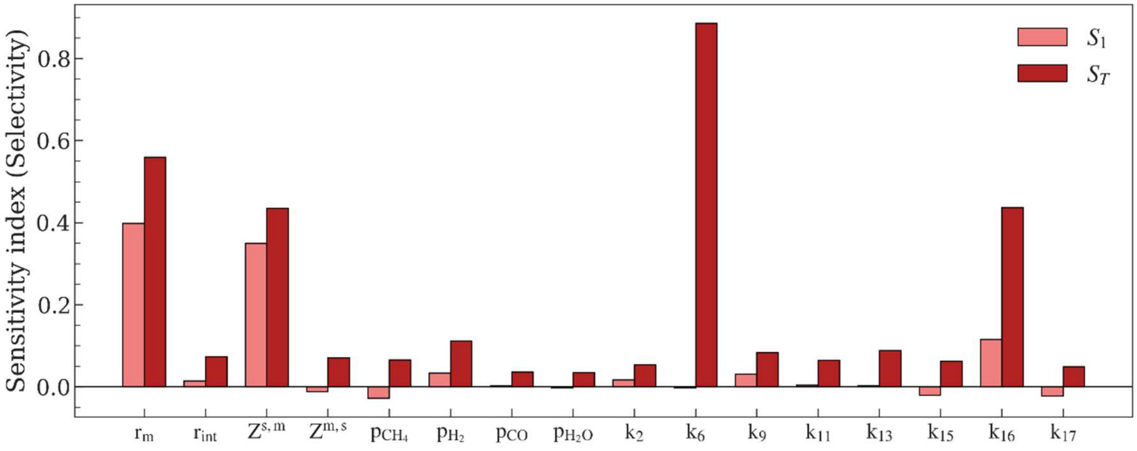

Figure S10. Sobol sensitivity indices of model parameters for DRM selectivity.

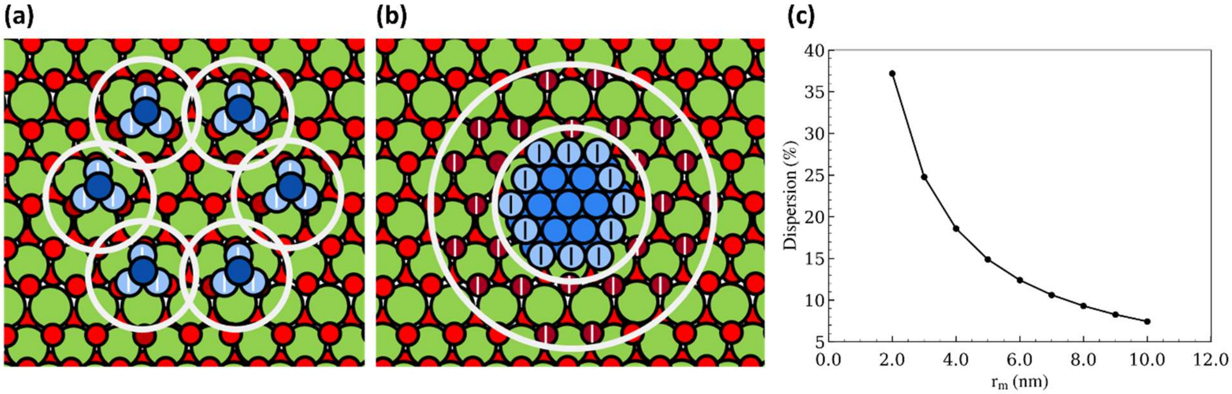

Figure S11. Schematic representation of **(a)** small and **(b)** large Ni nanoparticles deposited on $CeO_2(111)$. **(c)** Calculated dispersion for Ni nanoparticles as a function of r_m

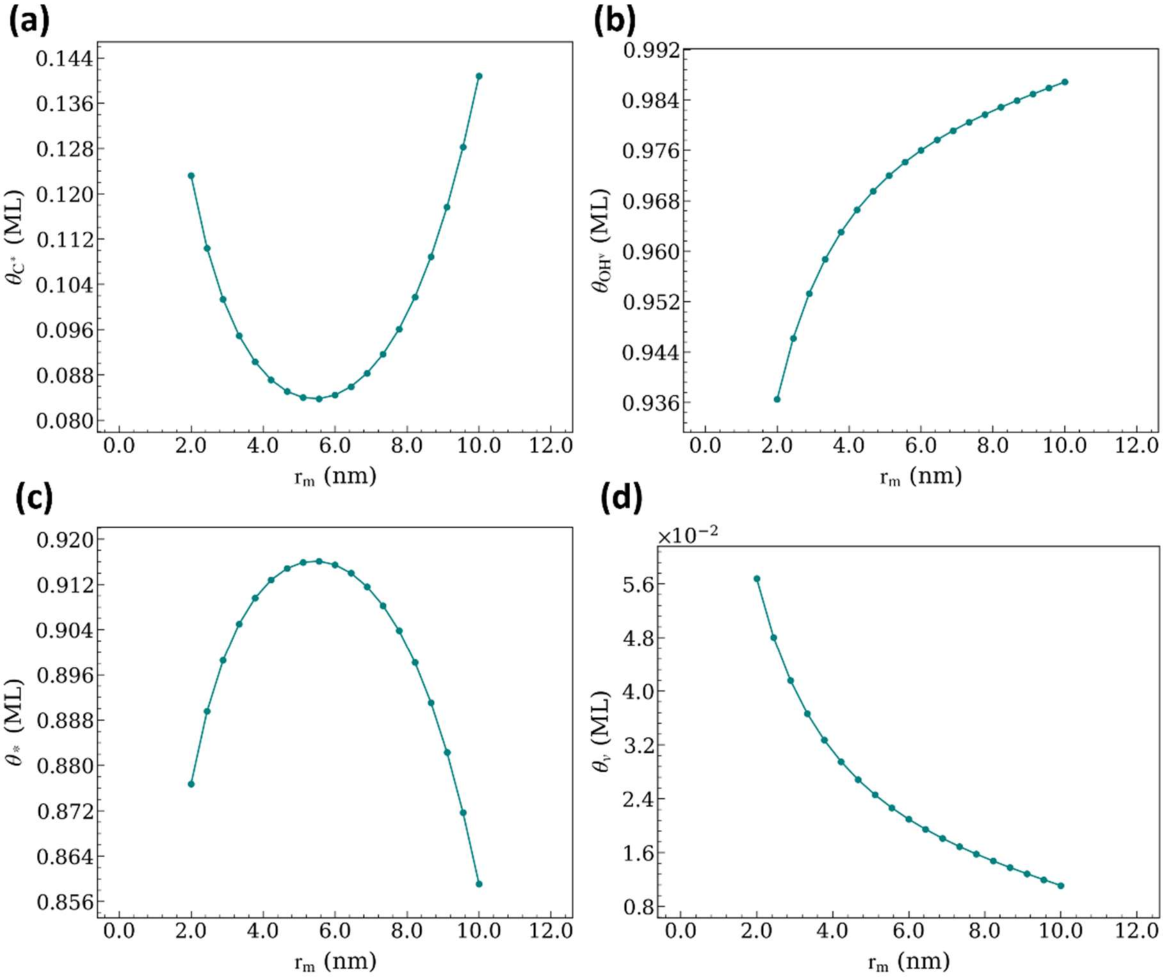

Figure S12. Steady state coverages of **(a)** C^*, **(b)** OH^v, **(c)** *, and **(d)** ^{v} species as a function of r_m at $p_{CH_4} = p_{CO_2} = 0.5$ bar ; $p_{CO} = p_{H_2} = p_{H_2O} = 0$; $T = 973.15$ K ; $r_{int} = 0.38$ nm

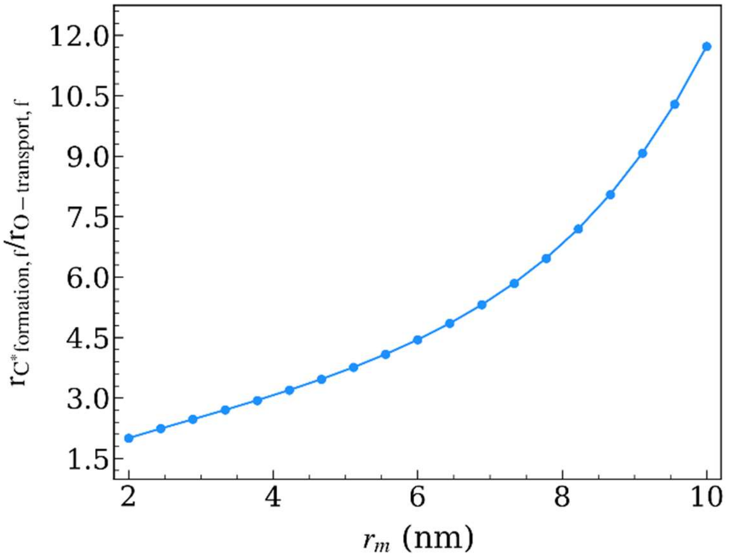

Figure S13. Ratio of forward rates of C^* formation to O-transport ($r_{C^*formation,f}/r_{O\text{-}transport,f}$) as a function of r_m at $p_{CH_4} = p_{CO_2} = 0.5$ bar; $p_{CO} = p_{H_2} = p_{H_2O} = 0$; $T = 973.15$ K ; $r_{int} = 0.38$ nm ; $r_m = 4$ nm

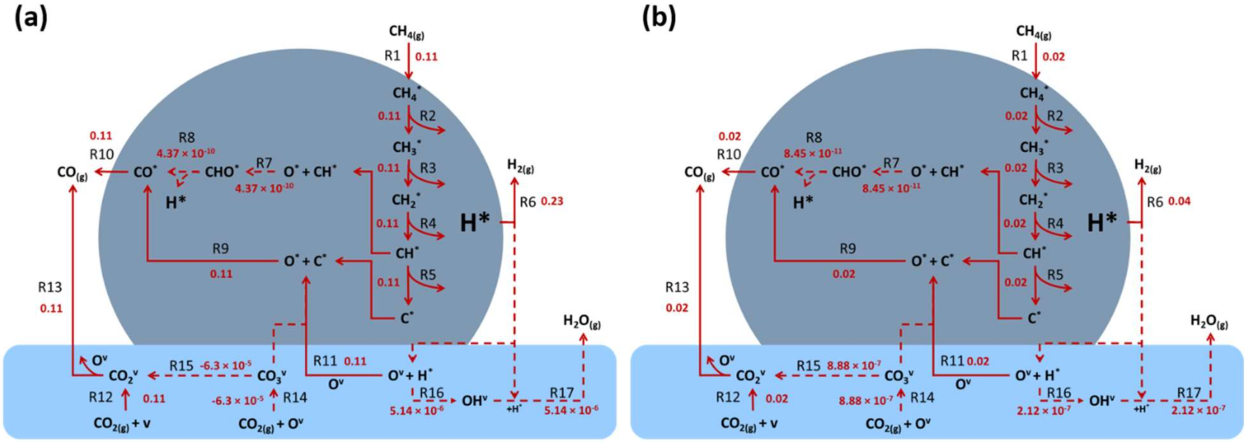

Figure S14. Reaction path analysis for **(a)** 2 nm Ni nanoparticle and **(b)** 10 nm Ni nanoparticle at $p_{CH_4} = p_{CO_2} = 0.5$ bar ; $p_{CO} = p_{H_2} = p_{H_2O} = 0$; $T = 973.15$ K ; $r_{int} = 0.38$ nm. Bolded and thinner arrows represent reactions that are part of the dominant and minor reaction pathways, respectively. All rates are expressed in mol g_{cat}^{-1} s^{-1}

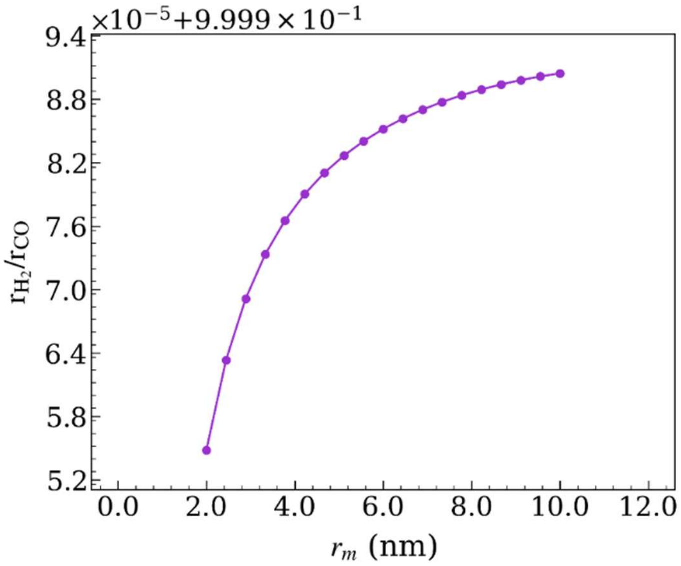

Figure S15. Selectivity as a function of r_m at $p_{CH_4} = p_{CO_2} = 0.5$ bar ; $p_{CO} = p_{H_2} = p_{H_2O} = 0$; $T = 973.15$ K ; $r_{int} = 0.38$ nm

References

(1) Lucas, J.; Padmanabha Naveen, N. S.; Janik, M. J.; Alexopoulos, K.; Noh, G.; Aireddy, D.; Ding, K.; Dorman, J. A.; Dooley, K. M. Improved Selectivity and Stability in Methane Dry Reforming by Atomic Layer Deposition on Ni-CeO $_2$ –ZrO $_2$ /Al $_2$ O $_3$ Catalysts. *ACS Catal.* **2024**, *14* (12), 9115–9133. https://doi.org/10.1021/acscatal.4c02019.

(2) Wang, T.; Chen, L.-Q.; Liu, Z.-K. Lattice Parameters and Local Lattice Distortions in Fcc-Ni Solutions. *Metall. Mater. Trans. A* **2007**, *38* (3), 562–569. https://doi.org/10.1007/s11661-007-9091-z.

(3) Moraes, P. I. R.; Bittencourt, A. F. B.; Andriani, K. F.; Da Silva, J. L. F. Theoretical Insights into Methane Activation on Transition-Metal Single-Atom Catalysts Supported on the CeO_2(111) Surface. *J. Phys. Chem. C* **2023**, *127* (33), 16357–16366. https://doi.org/10.1021/acs.jpcc.3c02653.

(4) Lustemberg, P. G.; Mao, Z.; Salcedo, A.; Irigoyen, B.; Ganduglia-Pirovano, M. V.; Campbell, C. T. Nature of the Active Sites on Ni/CeO_2 Catalysts for Methane Conversions. *ACS Catal.* **2021**, *11* (16), 10604–10613. https://doi.org/10.1021/acscatal.1c02154.

(5) Fujisaki, T.; Tsuji, Y.; Tu, P. H.; Doan, T. C. D.; Rivera Rocabado, D. S.; Staykov, A. T.; Yashiro, K.; Shiratori, Y. Investigating Ni Nanoparticles on CeO_2 for Methane Dissociation: A Comparative Study of Theoretical Calculations and Experimental Insights. *Phys. Chem. Chem. Phys.* **2025**, *27* (10), 5024–5036. https://doi.org/10.1039/d4cp01324g.

(6) Wu, C.; Xiao, Z.; Wang, L.; Li, G.; Zhang, X.; Wang, L. Modulating Oxidation State of Ni/CeO_2 Catalyst for Steam Methane Reforming: A Theoretical Prediction with Experimental Verification. *Catal. Sci. Technol.* **2021**, *11* (5), 1965–1973. https://doi.org/10.1039/d0cy02197k.

(7) Qu, P.-F.; Wang, G.-C. A Comprehensive Mechanistic Study for Dry Reforming of Methane over CeO_2-Supported TM_4 Clusters (TM = Ru, Pt, Co, Ni). *ACS Appl. Mater. Interfaces* **2024**, *16* (48), 66052–66065. https://doi.org/10.1021/acsami.4c13263.

(8) Singha, R. K.; Tsuji, Y.; Mahyuddin, M. H.; Yoshizawa, K. Methane Activation at the Metal–Support Interface of Ni_4–CeO_2(111) Catalyst: A Theoretical Study. *J. Phys. Chem. C* **2019**, *123* (15), 9788–9798. https://doi.org/10.1021/acs.jpcc.8b11973.

(9) Lian, Z.; Olanrele, S. O.; Si, C.; Yang, M.; Li, B. Critical Role of Interfacial Sites between Nickel and CeO_2 Support in Dry Reforming of Methane: Revisit of Reaction Mechanism and Origin of Stability. *J. Phys. Chem. C* **2020**, *124* (9), 5118–5124. https://doi.org/10.1021/acs.jpcc.9b09856.

(10) Wang, J.; Wang, G.-C. Methane Combustion Mechanisms on Ni_{10}/CeO_2 Studied by DFT, Microkinetic Modeling, and Kinetic Monte Carlo Simulation. *J. Phys. Chem. C* **2024**, *128* (31), 12978–12986. https://doi.org/10.1021/acs.jpcc.4c02579.

(11) Fan, C.; Zhu, Y.-A.; Yang, M.-L.; Sui, Z.-J.; Zhou, X.-G.; Chen, D. Density Functional Theory-Assisted Microkinetic Analysis of Methane Dry Reforming on Ni Catalyst. *Ind. Eng. Chem. Res.* **2015**, *54* (22), 5901–5913. https://doi.org/10.1021/acs.iecr.5b00563.

(12) Lustemberg, P. G.; Feria, L.; Ganduglia-Pirovano, M. V. Single Ni Sites Supported on CeO_2(111) Reveal Cooperative Effects in the Water–Gas Shift Reaction. *J. Phys. Chem. C* **2019**, *123* (13), 7749–7757. https://doi.org/10.1021/acs.jpcc.8b06231.

(13) Lozano-Reis, P.; Gamallo, P.; Sayós, R.; Illas, F. Comprehensive Density Functional and Kinetic Monte Carlo Study of CO_2 Hydrogenation on a Well-Defined Ni/CeO_2 Model Catalyst: Role of Eley–Rideal Reactions. *ACS Catal.* **2024**, *14* (4), 2284–2299. https://doi.org/10.1021/acscatal.3c05336.

(14) Liu, H.; Zhang, Y.; Liu, S.; Li, S.; Liu, G. Ni-CeO2 Nanocomposite with Enhanced Metal-Support Interaction for Effective Ammonia Decomposition to Hydrogen. *Chem. Eng. J.* **2023**, *473*, 145371. https://doi.org/10.1016/j.cej.2023.145371.

(15) Jiang, Y.; Wang, S.; Xu, J.; Zheng, M.; Yang, Y.; Wu, X.; Xia, C. Hydrogen Oxidation Pathway Over Ni–Ceria Electrode: Combined Study of DFT and Experiment. *Front. Chem.* **2021**, *8*. https://doi.org/10.3389/fchem.2020.591322.

(16) Alkhoori, A. A.; Elmutasim, O.; Dabbawala, A. A.; Vasiliades, M. A.; Petallidou, K. C.; Emwas, A.-H.; Anjum, D. H.; Singh, N.; Baker, M. A.; Charisiou, N. D.; Goula, M. A.; Efstathiou, A. M.; Polychronopoulou, K. Mechanistic Features of the CeO_2-Modified Ni/Al_2O_3 Catalysts for the CO_2 Methanation Reaction: Experimental and Ab Initio Studies. *ACS Appl. Energy Mater.* **2023**, *6* (16), 8550–8571. https://doi.org/10.1021/acsaem.3c01437.

(17) Lozano-Reis, P.; Prats, H.; Gamallo, P.; Illas, F.; Sayós, R. Multiscale Study of the Mechanism of Catalytic CO_2 Hydrogenation: Role of the Ni(111) Facets. *ACS Catal.* **2020**, *10* (15), 8077–8089. https://doi.org/10.1021/acscatal.0c01599.

(18) Salcedo, A.; Irigoyen, B. Unraveling the Origin of Ceria Activity in Water–Gas Shift by First-Principles Microkinetic Modeling. *J. Phys. Chem. C* **2020**, *124* (14), 7823–7834. https://doi.org/10.1021/acs.jpcc.0c00229.

(19) Yu, Y.; Xia, W.; Yu, A.; Simakov, D. S. A.; Ricardez-Sandoval, L. Transition-Metal-Doped CeO_2 for the Reverse Water-Gas Shift Reaction: An Experimental and Theoretical Study on CO_2 Adsorption and Surface Vacancy Effects. *ChemSusChem* **2025**, *18* (2). https://doi.org/10.1002/cssc.202400681.

(20) Kumari, N.; Haider, M. A.; Agarwal, M.; Sinha, N.; Basu, S. Role of Reduced CeO_2(110) Surface for CO_2 Reduction to CO and Methanol. *J. Phys. Chem. C* **2016**, *120* (30), 16626–16635. https://doi.org/10.1021/acs.jpcc.6b02860.

(21) Cheng, Z.; Sherman, B. J.; Lo, C. S. Carbon Dioxide Activation and Dissociation on Ceria (110): A Density Functional Theory Study. *J. Chem. Phys.* **2013**, *138* (1). https://doi.org/10.1063/1.4773248.

(22) Thomas C. Allison. NIST-JANAF Thermochemical Tables - SRD 13, 2013. https://doi.org/10.18434/T42S31.

(23) *The Microkinetics of Heterogeneous Catalysis*; Dumesic, J. A., Ed.; ACS professional reference book; American Chemical Society: Washington, DC, 1993.

(24) Foppa, L.; Margossian, T.; Kim, S. M.; Müller, C.; Copéret, C.; Larmier, K.; Comas-Vives, A. Contrasting the Role of Ni/Al_2O_3 Interfaces in Water–Gas Shift and Dry Reforming of Methane. *J. Am. Chem. Soc.* **2017**, *139* (47), 17128–17139. https://doi.org/10.1021/jacs.7b08984.

(25) Ren, B.; Li, J.; Wen, G.; Ricardez−Sandoval, L.; Croiset, E. First-Principles Based Microkinetic Modeling of CO_2 Reduction at the Ni/SDC Cathode of a Solid Oxide Electrolysis Cell. *J. Phys. Chem. C* **2018**, *122* (37), 21151–21161. https://doi.org/10.1021/acs.jpcc.8b05312.
